# Mouse p53-Deficient Cancer Models as Platforms for Obtaining Genomic Predictors of Human Cancer Clinical Outcomes

**DOI:** 10.1371/journal.pone.0042494

**Published:** 2012-08-07

**Authors:** Marta Dueñas, Mirentxu Santos, Juan F. Aranda, Concha Bielza, Ana B. Martínez-Cruz, Corina Lorz, Miquel Taron, Eva M. Ciruelos, José L. Rodríguez-Peralto, Miguel Martín, Pedro Larrañaga, Jubrail Dahabreh, George P. Stathopoulos, Rafael Rosell, Jesús M. Paramio, Ramón García-Escudero

**Affiliations:** 1 Molecular Oncology Unit, CIEMAT, Madrid, Spain; 2 Departamento de Inteligencia Artificial, Universidad Politécnica de Madrid, Boadilla del Monte, Madrid, Spain; 3 Catalan Institute of Oncology, Hospital Germans Trias i Pujol, Badalona, Spain; 4 Medical Oncology Department, Hospital Universitario 12 de Octubre, Madrid, Spain; 5 Pathology Department, Hospital Universitario 12 de Octubre, and Instituto de Investigación Hospital 12 de Octubre i+12, Universidad Complutense, Madrid, Spain; 6 Hospital General Gregorio Marañón, Universidad Complutense, Madrid, Spain; 7 Surgery Department, Athens Medical Center, Athens, Greece; 8 Oncology Department, Errikos Dunant Hospital, Athens, Greece; Thomas Jefferson University, United States of America

## Abstract

Mutations in the TP53 gene are very common in human cancers, and are associated with poor clinical outcome. Transgenic mouse models lacking the Trp53 gene or that express mutant Trp53 transgenes produce tumours with malignant features in many organs. We previously showed the transcriptome of a p53-deficient mouse skin carcinoma model to be similar to those of human cancers with TP53 mutations and associated with poor clinical outcomes. This report shows that much of the 682-gene signature of this murine skin carcinoma transcriptome is also present in breast and lung cancer mouse models in which p53 is inhibited. Further, we report validated gene-expression-based tests for predicting the clinical outcome of human breast and lung adenocarcinoma. It was found that human patients with cancer could be stratified based on the similarity of their transcriptome with the mouse skin carcinoma 682-gene signature. The results also provide new targets for the treatment of p53-defective tumours.

## Introduction

Mutations in the TP53 tumour suppressor gene are very common in human cancers, and in most cases are associated with a poor clinical outcome. Although great efforts have been made to find specific therapies for TP53-mutant cancers [1], none are currently used in the clinical setting. The lack of such therapies may be explained by the wide diversity of p53-related genomic alterations (point or truncating mutations, oncogenic or dominant-negative mutations, loss of heterozygosity, etc.) and by the presence of additional alterations in oncogenic signalling pathways [2]. Besides, such mutations are predictors of resistance to Nutlin-3a [3], an inhibitor of the MDM2 E3 ligase that negatively regulates p53 protein levels. However, the sensitivity of human cancer cell lines to chemotherapeutic drugs is not associated to p53 mutations [3]. The search for effective therapies for mutant patients is therefore of prime importance. One way of arriving at a treatment might be to identify and validate molecular biomarkers of TP53-based carcinogenesis, some of which might be suitable as targets for therapy. An added value of p53-based biomarkers would be their potential use in predicting the response to cancer therapies, thus allowing for the personalised treatment of patients.

There are different ways to search for correlations between tumour gene expression (GE) patterns and the clinical behaviour of tumours [4]. In the model-driven approach, the transcriptome of cells exposed to specific stimuli (such as a wound) or after the activation of specific oncogenic pathways, is used to determine a prognosis [5], [6]. This approach has the drawback that the experimental model used might not accurately reflect the processes that occur in tumours. The advantage, however, is that the model system acts as a “filter” of genes that are important in oncogenic signalling. The use of genetically engineered mouse models (GEMMs) designed to emulate the genetic alterations found in human cancers represents a great advance in this area. The targeted over-expression of a particular oncogene or knockout of a specific tumour suppressor gene in a well defined genetic background offers many advantages for studying tumour progression initiated by genetic aberrations [7]. A major benefit of GEMMs over cellular systems is that mouse carcinomas contain tumour cells as well as stromal and endothelial cells, which all contribute to a tumour’s biology [8]. Thus, genome-wide GE profiles of primary carcinomas from GEMMs of cancer [9], [10], as well as comparisons between metastatic and primary mouse carcinoma samples, have been used to try to develop predictors of the outcome of human cancer [11].

We previously reported that a 682-gene expression signature common to two skin carcinoma models lacking p53 (alone or combined with a lack of pRb, hereafter referred to as p53^ΔEC^ and p53^ΔEC^;pRb^ΔEC^ respectively) in stratified epithelia [12], [13] showed strong similarities to signatures of human primary carcinomas involving TP53 mutations (both truncating and point) arising in different anatomical locations. Bioinformatic tools used to examine the mouse skin carcinoma gene signature and transcriptomes of different types of human cancer showed a human signature of 20 overexpressed genes associated with TP53 mutation and a poor prognosis. Importantly, when patients with cancer were stratified depending on the expression of these genes, different clinical outcomes were observed: the stronger the expression, the lower the probability of surviving cancers such as breast carcinoma (BC) or multiple myeloma [12].

This report shows the above 682-gene signature to be present in different GEMMs of BC and lung adenocarcinoma (LAd). Importantly, the similarities were strongest in those models involving p53 inhibition, and in the metastatic samples arising from some of them. Using this 682-gene signature, we obtained and validated GE tests able to stratify patients with these cancers into groups with significant differences in expected clinical outcome, and which showed high sensitivity in terms of the identification of patients with a potentially good outcome.

## Results

### The 682-gene Signature is Present in GEMMs of BC and LAd with p53 Inhibition

Genome-wide microarray analyses have shown human aggressive and/or TP53-mutant tumours to possess transcriptomes resembling the 682-gene mouse skin carcinoma signature [12]. These similarities are particularly noticeable for human BC and LAd [12]. Further, the transcriptome of the mouse skin carcinomas shows strong similarities to that of embryonic stem cells (ESC), suggesting that p53 deficiency induces a potent de-differentiation process in epithelial cells [12]. p53-mutant human BCs show these ESC signatures too [14]. This is in agreement with the locally invasive properties of these mouse tumours, and their propensity to metastasise to distant organs [15].

**Figure 1:**
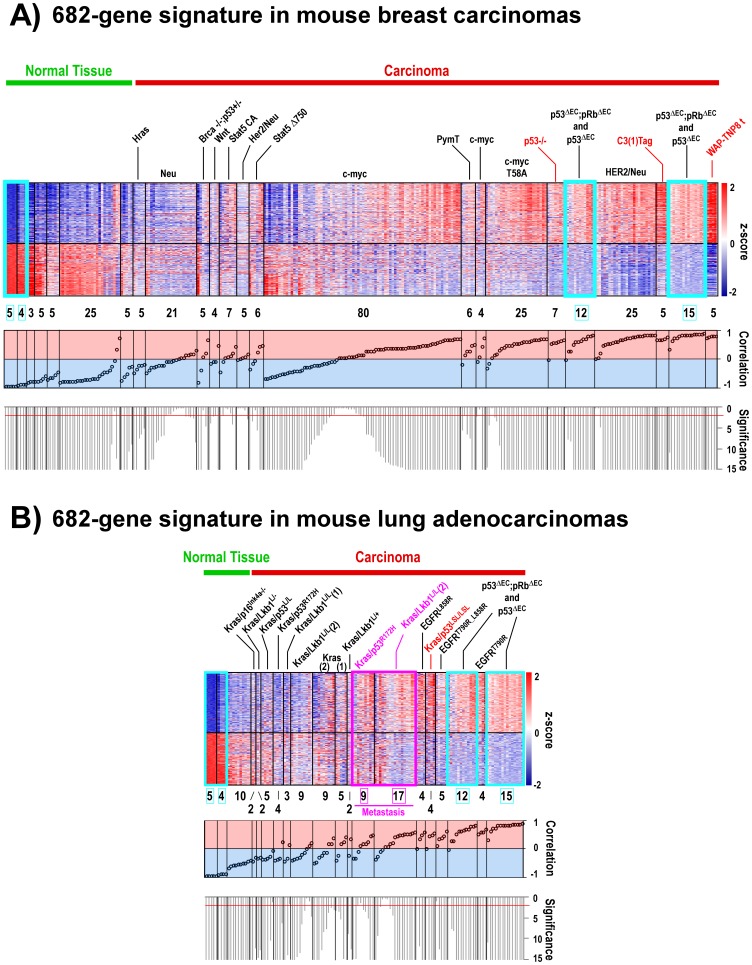
The mouse skin 682-gene signature is significantly present in mouse mammary and lung carcinoma models showing p53 inhibition. Heatmaps of the 682-gene signature transcripts from (**A**) primary breast carcinomas and normal mammary glands from different transgenic GEMMs (upper panel), and from (**B**) primary and metastatic lung adenocarcinomas and normal lungs from different transgenic GEMMs (upper panel) (Table S1) are shown. The T-values returned by Student’s t-test comparisons between normal skin and carcinoma samples in which the 682-gene signature was determined (GSE11990) were used to build a centroid template. The Pearson correlation coefficient (and the corresponding p-value) with respect to the centroid was calculated for each mouse sample. Samples were ordered from left to right based on increasing correlation. Probesets are ordered from top to bottom based on T-values (see Materials and Methods). Samples within blue rectangles are normal skin samples and skin tumour samples. The number of samples in each group is shown under the heatmaps. Pearson values are shown in the middle panel. Values range from −1 (negative correlation, bluish background) to +1 (positive correlation, reddish background). The significance value for the correlation is shown in the lower panel as –log_10_(p-val). The red line indicates p-val = 0.01. Genotypes highlighted in red are models with p53 alterations significantly correlated with the 682-signature. Samples highlighted in pink are metastases. In (**B**), the Kras (1) and Kras/Lkb1^L/L^ (1) samples are from the GSE6135 dataset; the Kras (2) and Kras/Lkb1^L/L^ (2) samples are from the GSE21581 dataset.

Given the significant GE similarities between these mouse skin tumours and human BC and LAd with a p53 mutation, in the present work the 682-gene signature was sought in GEMMs of BC and LAd showing p53 inhibition. Raw GE data were downloaded from the GEO database (Table S1) [10], [11], [16]–[22] and similarities with the 682-gene tumour signature sought by calculating Pearson correlations (see Materials and Methods). Metagenomic comparisons showed carcinomas from specific BC (Fig. 1A) and LAd (Fig. 1B) GEMMs to have GE profiles very similar to those of mouse skin carcinoma. With respect to BC, models of p53 inactivation via the expression of the SV40 large T-antigen (C3(1)Tag and WAP-TNP8 models) [20], [23], and the p53^fl/fl^;MMTV-cre transplant model [23], were among the most similar (highlighted in red, Fig. 1A). Significant similarities were seen with the 682-gene signature for a LAd model in which p53 expression is repressed in the presence of an oncogenic Kras^G12D^ allele (Kras^LA2/+^;Trp53^LSL/LSL^;Rosa26Cre^ERT2^ model) [18] (highlighted in red, Fig. 1B). Importantly, the p53-deficient skin carcinomas shared GE patterns with metastatic samples arising in a Kras/p53^R172H^ and a Kras/Lkb1^L/L^ LAd GEMM [10], [11], confirming their aggressive molecular properties (highlighted in pink, Fig. 1B). Importantly, most Kras/p53^R172H^ metastatic samples lose the wild type (WT) Trp53 allele during malignant transformation [10]. These comparisons between GEMMs show that the 682-gene skin signature is significantly present in p53-deficient mouse lung and mammary carcinomas, and might be considered a common signature of p53-deficient carcinoma GEMMs.

**Figure 2:**
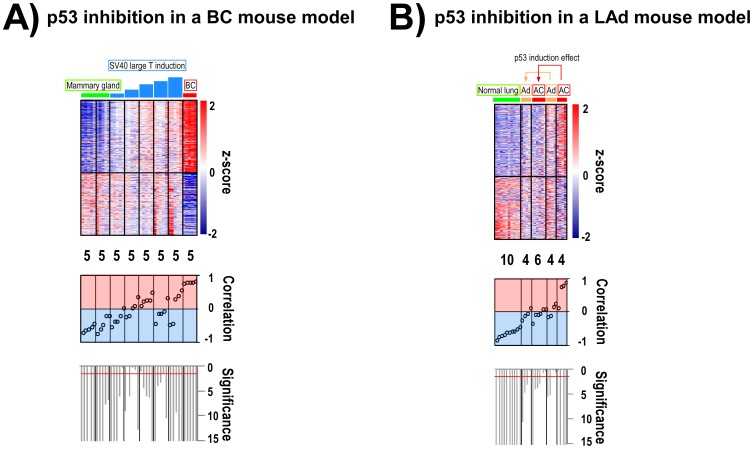
The 682-gene signature expression pattern in mouse carcinomas is dependent on p53 expression. **A**) SV40 Large-T antigen expression in mammary gland was analysed at various time-points during carcinoma formation in transgenic WAP-TNP8 mice. Heatmaps of 682-gene signature transcripts from normal mammary glands (green), primary breast carcinomas (red) and mammary samples with transgene expression at 1, 2, 3, 4 and 5 months (blue) are shown (upper panel). **B**) p53 expression was induced in lung adenomas and adenocarcinomas in the Kras^LA2/+^;Trp53^LSL/LSL^;Rosa26Cre^ERT2^ mouse model. The heatmaps of the 682-gene signature transcripts from normal lungs (green), lung adenomas (orange) and adenocarcinomas (red) (treated and untreated) are shown (upper panel). In **A** and **B**, sample groups are ordered from left to right based on increasing Pearson correlation with the centroid template based on the 682-gene signature. Probesets are ordered from top to bottom based on T-values (see Materials and Methods). The number of samples in each group is shown under the heatmap. The correlation values for individual samples with the centroid are shown in the middle panel. Values range from −1 (negative correlation, bluish background) to +1 (positive correlation, reddish background). The significance of the correlation for each sample is shown in the lower panel as –log_10_(p-val). The red line indicates a p-val of 0.01.

Since the p53-deficient primary skin samples profiled were overt carcinomas, it cannot be ruled out that other oncogenic events may be acting as major players in their transcriptome deregulation, and therefore in the similarities seen with human primary tumours with poor outcome. To detect any direct implication of p53 protein activity in the GE pattern, breast and lung GEMMs in which p53 expression levels could be modulated were examined. In the WAP-TNP8 model, time-course analyses of p53 inhibition by means of SV40 large T-antigen expression (1, 2, 3, 4 and 5 months) showed a progressive increase in the overexpression of already overexpressed (plus a reduction in the expression of already underexpressed) 682-signature genes in mammary carcinomas (Fig. 2A). In addition, the restoration of Trp53 expression with tamoxifen in Kras^LA2/+^;Trp53^LSL/LSL^;Rosa26Cre^ERT2^ mouse lung adenomas and adenocarcinomas reduced the overexpression (and induced the underexpression) of 682-signature mRNAs (Fig. 2B). As previously reported [18], tamoxifen-dependent p53 induction in these malignant lung adenocarcinomas leads to significant tumour cell loss. These results directly associate tumour reduction (upon p53 expression) with the disappearance of the 682-gene signature, indicating that its transcriptional regulation is dependent on p53. This confirms that this signature is common to both p53-altered human and mouse carcinomas.

### Development and Validation of a Prognostic Genomic Test for Human BC Clinical Outcome

Given the similarities between the mouse skin signature and those of mouse lung and BC (see above) and human tumours arising in these organs [12], the question arose as to whether the 682-gene signature could be used to develop prognostic tests for these human cancers. To develop such genomic predictors, the rodent signature was combined with GE data for primary human BC or LAd samples with known survival data.

**Figure 3:**
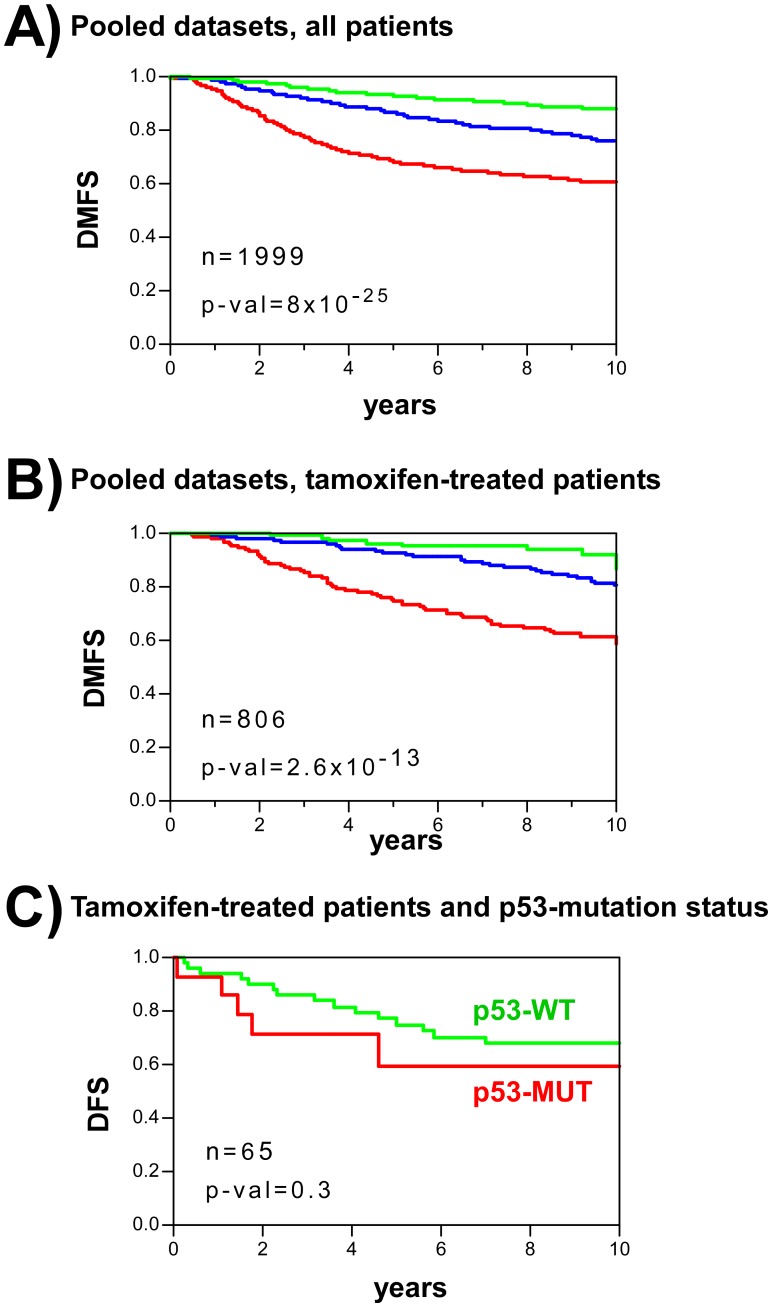
Human BC patient stratification using the mouse-derived 40-gene predictor test. **A**) Kaplan-Meier curves of distant metastasis-free survival (DMFS) for a pooled population of 12 GE datasets of patients with BC. Patients were stratified based on the 40-gene test as of low (green), intermediate (blue) or high (red) risk (see Materials and Methods). **B**) Kaplan-Meier curves of DMFS from ER+, tamoxifen-treated women with BC. Patients were stratified based on the 40-gene test as of low (green), intermediate (blue) or high (red) risk. **C**) Kaplan-Meier curves for ER+, tamoxifen-treated patients with breast cancer in the Miller dataset. Patients were stratified depending on the presence (red) or absence (green) of p53 mutations. p-val: significance of survival differences (log-rank test).

For human BC, a subgroup of 40 probesets, corresponding to 32 genes (40-gene test), was selected based on optimal distant metastasis prediction accuracy and small gene set size (Materials and Methods, Figs. S1 and S2A-C, Tables S2 and S3). The 40-gene test stratified BC patients into three risk groups: high, intermediate and low. The prediction accuracy of the test was validated in 12 additional datasets, comprising a total of 2993 tumour samples, 4 different endpoints, and 2 microarray platforms (Affymetrix and Agilent) (Fig. 3A, Figs. S3 and S4, Table S2). Multivariate Cox regression analysis including both genomic and clinical variables showed the 40-gene test to discriminate patient risk groups independent of clinical prognostic factors (Table 1).

**Table 1:** Multivariate Cox regression including the 40-gene test and breast cancer clinical variables.

Variable		HR^1^	95% CI^2^	p-val
40-gene test	H *vs* L	4.43	2.46 to 7.95	8.5×10^−8^ *
	I *vs* L	2.06	1.13 to 3.74	6.7×10^−7^ *
tumor size (T2 *vs* T1)		2.21	1.52 to 3.21	3.0×10^−5^ *
Node (positive *vs* negative)		1.07	0.78 to 1.46	0.69
ER status (negative *vs* positive)		1.15	0.77 to 1.72	0.50
Grade (3 *vs* 1 and 2)		1.00	0.72 to 1.41	0.98
Age (≤50 *vs* >50, years)		1.27	0.82 to 1.97	0.28

Total number of samples is 668. Endpoint analyzed was distant metastasis at 10 years.

Cox analysis was done stratifying by dataset.

1 HR: Hazard ratio.

2 CI: confidence interval.

* Significant p-values.

Most breast cancers are oestrogen receptor positive (ER+) and are treated with adjuvant hormonal therapy, such as tamoxifen. Interestingly, although the 40-gene test was developed using data from patients that received no such treatment, it predicted the outcome for such hormonally-treated patients as well (Fig. 3B). A possible explanation for this is that this test identifies tumours with inherent malignant behavior, and which are therefore less prone to respond to adjuvant therapy. Alternatively, it may be that high risk patients with BC suffer inhibition of the p53-dependent pathway linked to ER signalling pathways [24]–[27]. In agreement with this hypothesis it should be noted that a reduced response to tamoxifen has been reported in patients with BC carrying TP53 mutations [28], [29] (Fig. 3C).

### Development and Validation of a Prognostic Genomic-clinical Test for Human LAd Clinical Outcome

**Figure 4:**
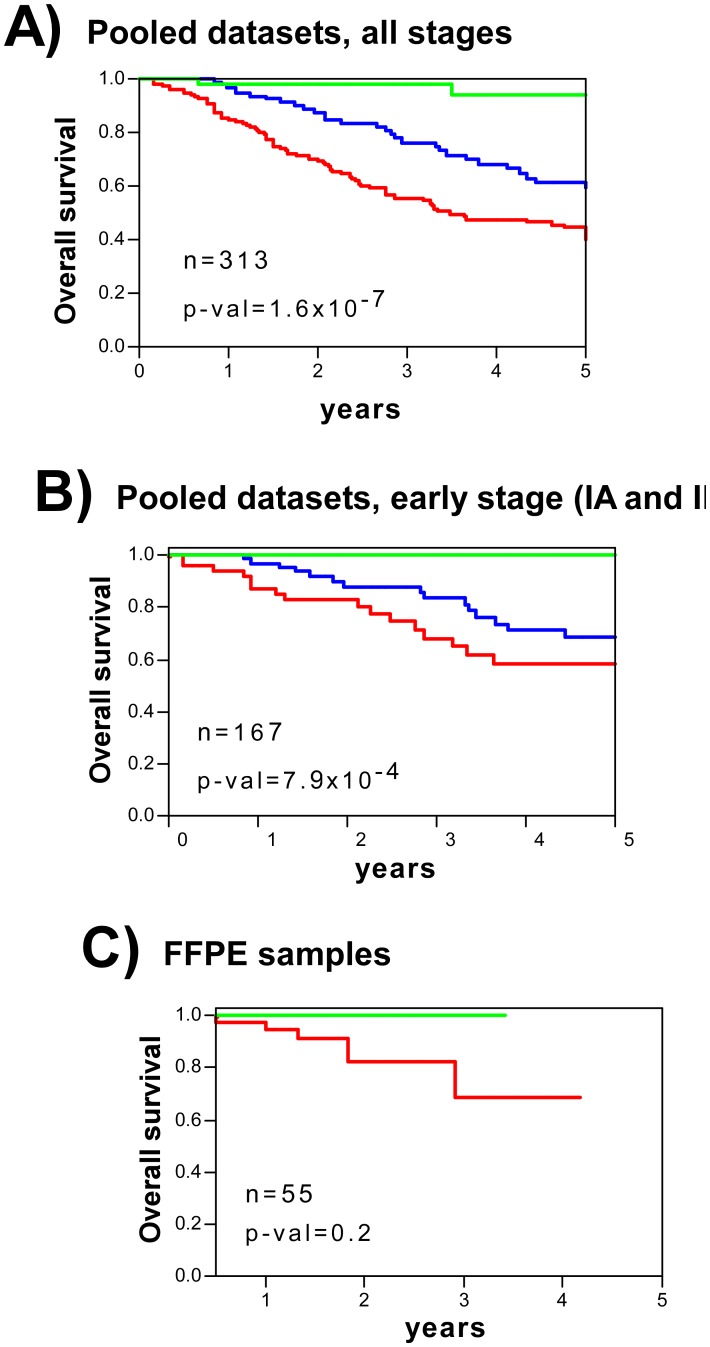
Human LAd patient stratification using the mouse-derived 36-gene genomic-clinical predictor test. **A**) Kaplan-Meier curves for overall survival (OS) for the pooled population of patients with lung cancer in three datasets including patients with all disease stages. Patients were stratified based on the 36-gene test as of low (green), intermediate (blue) or high (red) risk (see Materials and Methods). **B**) Kaplan-Meier curves for early stage patients (Stages IA and IB). Patients were stratified based on the 36-gene test as of low (green), intermediate (blue) or high (red) risk. **C**) Kaplan-Meier curves for patients profiled using qRT-PCR and FFPE samples. Patients were stratified based on the 36-gene test as of low (green), or high-intermediate (red) risks (see Materials and Methods). Owing to the small sample size, the intermediate and high risk groups were pooled. p-val: significance of survival differences (log-rank test).

Using the same approach used with BC, an optimal group of 36 probesets corresponding to 30 genes (36-gene test) was obtained to predict overall survival (Materials and Methods, Fig. S1, Fig. S2 [panels A, D and E], Tables S4 and S5). Shedden et al. [30] reported that the accuracy of genomic predictors of LAd outcome could be improved by incorporating certain clinical variables. Thus, a clinical predictor test was developed including tumour stage, patient gender and age (Fig. S5A). The combination of both genomic and clinical information (36-gene genomic-clinical test) increased the prediction accuracy, of overall survival, allowing patients to be stratified into three risk groups (low, intermediate and high) using the same approach as for BC. Validation in 3 external microarray GE datasets showed the accuracy of the combined test with the pooled patients (n = 313) (Fig. 4A), or in individual datasets (Fig. S6). More importantly, it also accurately predicted clinical outcome among early stage patients (Fig. 4B, Fig. S6). As the number of reported human LAd samples that we have used for validation is lower when compared to human BC, we decided to add new LAd samples by performing GE from FFPE tumour blocks. This analysis would also aid to demonstrate the feasibility of the 36-gene genomic-clinical predictor using FFPE tissue. Validation was performed using quantitative real-time PCR (qRT-PCR) (Materials and Methods, Fig. S5B). The results confirmed that the genomic-clinical test stratified patients with different survival probabilities (Fig. 4C) with similar accuracy to that seen for ‘fresh’ (i.e., non-FFPE) samples profiled using GE microarrays (area under the curve [AUC] = 0.72, p-val = 1.4×10^−9^ for microarrays; AUC = 0.70, p-val = 0.05 for qRT-PCR). Univariate Cox regression analysis including all patients in the validation datasets (n = 362) showed significant risk differences between patient strata. The hazard ratio (HR) for OS at 5 years was 14.14 times higher (95% CI = 3.46 to 57.83, p-val = 0.0002) than in the high than the low risk groups. In addition, the hazard ratio (HR) for OS at 5 years was 7.60 times higher (95% CI 1.82 to 31.78, p-val = 0.005) for the high risk group than the intermediate risk group.

### Correlation between 40-gene Test and TP53 Mutations

Using metagenomic comparisons of GEMMs (Fig. 2), the time-course inhibition of p53 was seen to involve the progressive appearance of the 682-gene signature with BC formation. In addition, p53 restoration in mouse lung adenomas and adenocarcinomas led to the disappearance of the signature; other authors have reported tumour cell loss to occur as well [18]. A similar result was obtained for the 40-gene signature in the BC model, and for the 36-gene signature in the LAd model (Fig. S7). These findings support the idea of a major role for p53 in the control of the genes in both signatures. Network analyses of the 40-gene and 36-gene proteins in relation to p53 and pRb (since the 682-signature was obtained from the common transcriptomes of the p53^ΔEC^ and p53^ΔEC^;pRb^ΔEC^ models [12]) showed both p53 and pRb to be direct regulators of most of these proteins (Fig. S8A and C). Further, these signature genes appear to be important regulators of processes involved in carcinogenesis such as apoptosis, differentiation and proliferation (Fig. S8B and D).

**Figure 5:**
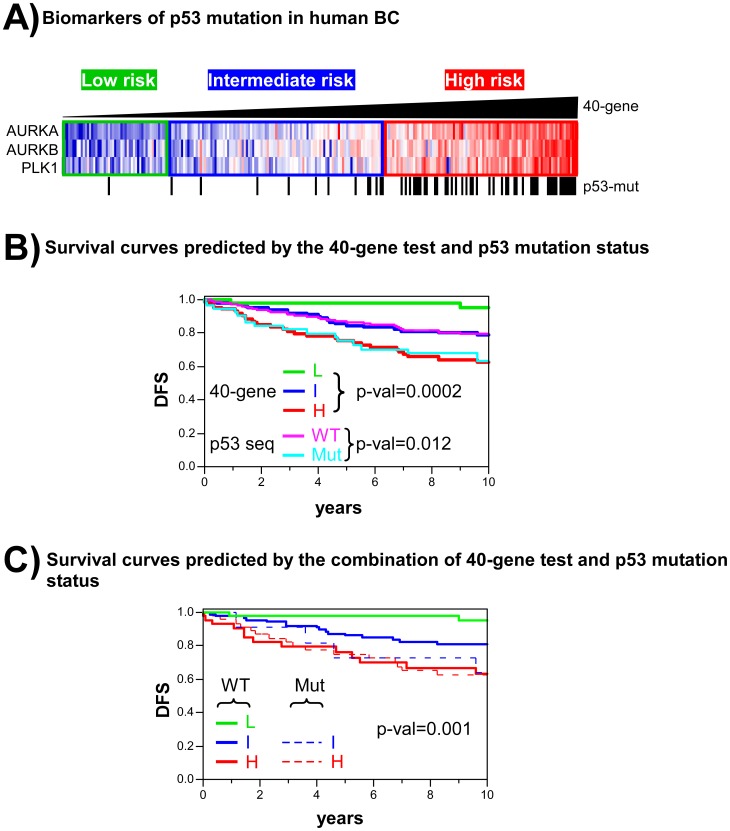
Comparison between the 40-gene test and p53 mutation status in terms of predicting the outcome of BC. **A**) The AURKA, AURKB and PLK1 genes within the 40-gene test are overexpressed in human BC with p53 mutations. Tumour samples were ordered by p53 Risk Score as determined by the 40-gene test; risk groups are shown as low (green), intermediate (blue) or high (red) risk. Note the existence of high risk tumours without p53 mutations. **B**) Comparison of patient stratification determined using the 40-gene test and p53 mutation status in the Miller BC dataset. The survival curves of both stratification methods are shown simultaneously for the same patient dataset. p-val: significance of survival differences (log-rank test). **C**) Combination of the 40-gene test and p53 mutation status for stratifying patients with BC. Patients are grouped as p53-WT (L, I and high risk groups) or p53-MUT (low, intermediate and high risk groups). Only one sample out of 251 was classified as of low risk and p53-MUT; this was not included in the graph. p-val: significance of survival differences (log-rank test).

The calculation of the risk score for the BC and LAd patients was based on the GE profiles of the p53-deficient tumours, not on the presence/absence of p53 mutations in sample patients as previously reported for BC predictors [28], [31]. Given the importance of p53 alterations in the appearance of human cancer, great effort has been directed towards the development of therapies that restore p53 function [1]. However, no such treatments are yet available in the clinical setting. Another possibility is to identify molecular biomarkers associated with p53 alterations that offer themselves as therapeutic targets. To examine this, we selected genes that are overexpressed in p53-mutant human BC tumours (Miller dataset, Table S2) [28], and for which specific inhibitors are in preclinical testing: AURKA, AURKB and PLK1 (Fig. 5A). These inhibitors, if validated clinically, might be usable for the treatment of patients with p53 mutations. Importantly, the overexpression of the AURKA, AURKB and PLK1 genes was also observed in non-p53 mutant tumours within the high risk group as assessed by the 40-gene test (Fig. 5A), showing that some patients with poor outcome suffering p53-WT tumours may also benefit from such therapies. To search for any potential anti-tumoral effect of these inhibitors in tumour samples with p53 deficiency, the GE profiles of human cancer cell lines and xenografts sensitive to targeting therapies were compared to the 682-gene signature. The similarities observed indicate their potential susceptibility to these agents. The human cancer xenografts that responded to AURKA inhibitors were found to be more similar to the mouse p53-deficient tumours than those that did not respond (Fig. S9A) [32]. Further, those cell lines sensitive to targeted therapies against AURKB and PLK showed strong similarities to the p53-deficient mouse carcinomas (Fig. S9B) [33]. Importantly, these sensitive cell lines included not only BC and LAd cell lines, but cells of other organs, suggesting an effect of these inhibitors in different cancer types. Another approach to search for targeted therapies that might be useful in p53-deficient tumours was performed using the Connectivity Map resource [34] (Materials and Methods). Briefly, we search for small molecule bioactive compounds (dubbed perturbagens) able to induce GE profiles with the reverse pattern of that observed in the 682-signature, so that they could be used to treat p53-deficient tumours. The results indicate that inhibitors of histone deacetylases (such as trichostatin A or vorinostat) are between the most significant perturbagens that may repress the 682-signature pattern (Table 2). Interestingly, the antipsychotic drug thioridazine also represses the p53-deficient carcinoma GE profiles, in line with recent evidences demonstrating that the drug antagonizes dopamine receptors that are expressed on cancer stem cells and on breast cancer cells [35].

**Table 2:** Top 10 pertubagens identified through the Connectivity Map that induce a reverse 682-signature.

Rank^1^	Perturbagen - cell line	Mean^2^	Description	p-val^3^
1	trichostatin A - PC3	−0.676	HDAC inhibitor	0
2	trichostatin A - MCF7	−0.600	HDAC inhibitor	0
3	vorinostat - MCF7	−0.708	HDAC inhibitor	0.00006
5	0175029-0000 - PC3	−0.763	nd	0.00048
6	sirolimus - MCF7	−0.389	mTOR inhibitor	0.00066
7	ellipticine - MCF7	−0.848	Topoisomerase IIinhibitor	0.0007
8	LY-294002- MCF7	−0.499	PIK3CA inhibitor	0.00084
9	thioridazine - PC3	−0.695	Dopamine receptor inhibitor	0.00118
15	harmine - MCF7	−0.842	Monoamine oxidase inhibitor	0.00233
16	tomatidine - MCF7	−0.830	ACAT1 inhibitor	0.00243

1 Ranking based upon permutation analysis of the same perturbagen made in the same cell line.

2 Arithmetic mean of the connectivity scores.

3 An estimate of the likelihood that the enrichment of a set of instances in the list of all instances in a given result would be observed by chance.

A comparison between clinical outcome as predicted by the 40-gene test and p53 mutation status was performed using the Miller dataset. The genomic test showed greater sensitivity than the p53 mutation status in terms of predicting patients with a good prognosis (see comparisons of the low risk [L, green line] and p53-WT [pink line] groups; Fig. 5B). Interestingly, patients without TP53 mutations but predicted to be at high risk by the 40-gene test showed poor survival potential (high risk and WT in Fig. 5C, red line). Importantly, these WT patients showed similar survival probabilities to the high risk TP53-mutant patients (high risk and MUT in Fig. 5C, dashed red line). A similar result was obtained when comparing the 40-gene test with the Miller GE-based predictor of p53 mutation status [28] (Fig. S10). Multivariate Cox regression including both predictors showed the results of the 40-gene test to be better correlated with survival than the p53 mutation genomic predictor (Table S6). These results indicate that the prediction of clinical outcome based on the 40-gene test to be more accurate than the TP53 mutation status, the consequence of its ability to detect poor outcome patients with no mutation and to discriminate low risk patients with greater sensitivity.

### p53 Dysfunction in Molecular Subtypes of Human BC and LAd

**Figure 6:**
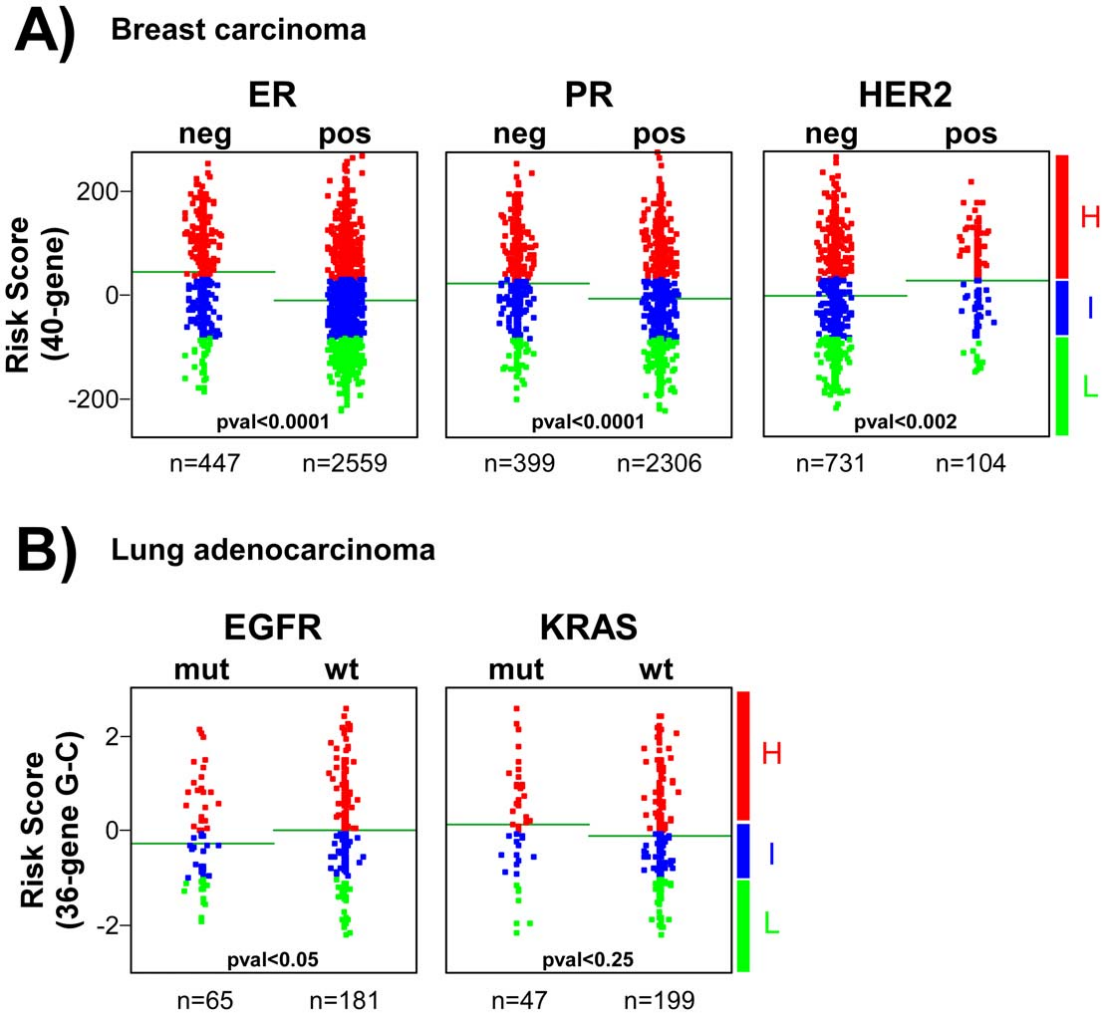
p53 dysfunction in molecular subtypes of human BC and LAd. Patient risk scores (p53RS) are represented depending on ER, PR and HER2 status using the 40-gene test for breast cancer patients (**A**) and depending on EGFR and KRAS mutation status as calculated by the 36-gene genomic-clinic test for lung adenocarcinoma patients (**B**). Each dot represents an individual sample value. Horizontal green lines represent mean values in each sample group. Student’s Ttest analysis was done to find significant differences in score values between patient biomarker subgroups (threshold p-val<0.05). Patients were stratified based on the risk groups as of low (green), or high-intermediate (red) risks (see Materials and Methods).

**Figure 7:**
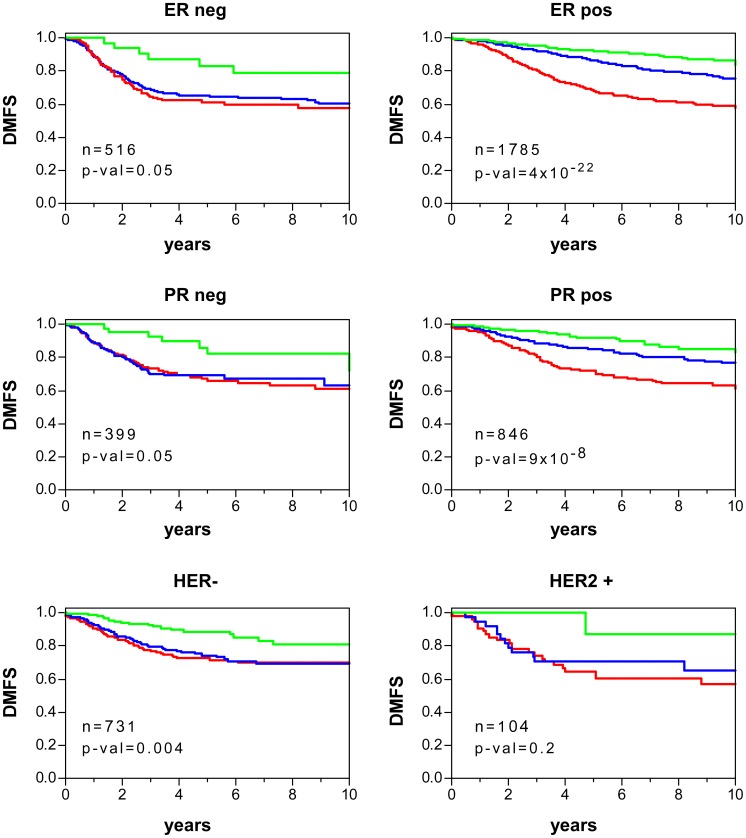
Survival curves of human BC stratified using 40-gene test and depending on molecular subtypes. Kaplan-Meier curves of distant metastasis-free survival (DMFS) for patients with BC depending on ER, PR and HER2 status. Patients were stratified based on the 40-gene test as of low (green), intermediate (blue) or high (red) risk (see Materials and Methods). p-val: significance of survival differences (log-rank test).

**Figure 8:**
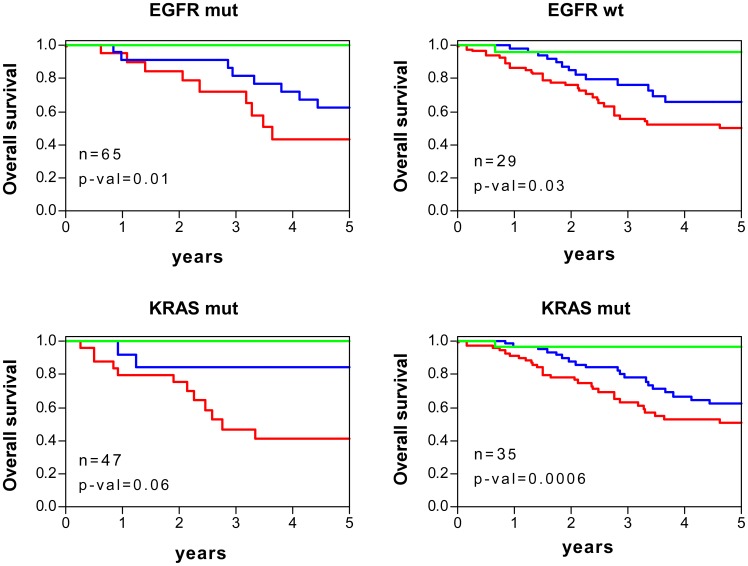
Survival curves of human LAd stratified using 36-gene genomic-clinic test and depending on molecular subtypes. Kaplan-Meier curves of overall survival for patients with LAd depending on EGFR and KRAS mutation status. Patients were stratified based on the 36-gene genomic-clinic test as of low (green), intermediate (blue) or high (red) risk (see Materials and Methods). p-val: significance of survival differences (log-rank test).

Currently, there are oncogene biomarkers defining molecular subtypes with different clinical outcome and/or targeted therapies in BC and LAd, as we have already mentioned for oestrogen receptor and breast cancer (see Fig. 3). The p53 dysfunction was analysed in these molecular subtypes by comparing the p53RS-derived values using the 40-gene test and 36-gene genomic-clinical test. For breast tumours, ER or progesterone receptor (PR) negative samples displayed higher risk score values than the positive ones, in line with their highest aggressive behavior (Fig. 6A) (Table S9). HER2-positive carcinomas exhibited higher score values (Fig. 6A), also in agreement with worse clinical outcome. For LAd, EGFR-mutant tumours showed lower risk score values (Fig. 6B) (Table S10), as expected due to their best clinical behavior. However, no significant differences were found between samples with or without KRAS mutations. Despite the mean differences in p53RS values, both 40-gene test (Fig. 7) and 36-gene genomic-clinical test (Fig. 8) stratified patients with significant survival differences independent on oncogene biomarker subgrouping.

## Discussion

The p53 pathway is one of the most important tumour suppression mechanisms; mutations affecting it are commonly found in the majority of cancer types. The correlation between such mutations and tumour malignancy, suggests the need for more detailed characterization of this pathway. High throughput technologies such as genome-wide GE analysis or next generation sequencing (NGS) may help to determine the alterations in individual tumours, which would allow personalized treatments and ultimately improve the care that could be offered to patients. However, arriving at effective personalized medicine depends on the availability of appropriate analysis model systems and adequate clinical evaluation/validation. The present work discusses a p53-deficient tumour mouse model system with molecular features leading to tumour aggressiveness, and the development and validation of GE signatures that can predict clinical outcomes in human BC and LAd. The results show the genes making up these signatures to be surrogate markers of p53-dependent pathway alterations, and possible candidates for targeting therapies.

We previously reported a mouse 682-gene signature seen in p53-deficient skin tumours to show significant molecular similarities to human cancer transcriptomes (such as those of BC and LAd) involving TP53 mutations and/or poor outcome. The present results show that such similarities are also present in GEMMs of BC and LAd carcinoma in which p53 expression or function is inhibited, confirming our previous findings. They also show that human and mouse carcinomas arising in different organs such as skin, lung and breast show strong similarities upon p53 alteration. Similar findings were reported by Deeb et al. [9], which identified a gene signature associated with clinical outcome of human BC, LAd and prostate cancer using GEMMs expressing SV40 T/t antigens. An explanation for the similarities in molecular profile between tumours of different organs may be that p53 inhibition induces an overall process of de-differentiation, giving rise to an ESC-like phenotype. This would agree with our previous results showing ESC signatures in the mouse skin carcinomas [12], with findings showing that tumour aggressiveness is predicted by these ESC GE profiles [36], and with the presence of such profiles in p53-mutant human BC tumours [14]. The observation that p53 inhibition in different organs induces a common GE program associated with poor clinical outcome also reinforces the direct role of p53 protein in the suppression of malignancy. Similarly, the present results show that time-dependent inhibition of p53 in a BC model or restoration of p53 expression in tumours in a LAd model is significantly correlated with the 682-, 40-, and 36-gene signatures *in vivo* (Fig. 2 and S7). Pathway analysis showed that p53 directly inhibits the genes overexpressed in the 40-gene and 36-gene signatures. Collectively these findings strengthen and support the major roles of p53 in multiple tissues of different organisms, and demonstrate that these gene signatures are surrogate biomarkers of p53 inhibition during carcinoma progression. Using the Oncomine database, the analysis of the transcriptome of human cell lines and xenografts with sensitivity to drugs designed against AURKA, AURKB or PLK1 kinases show a profile similar to that seen in the described mouse skin carcinomas. These similarities indicate that tumours with such profiles may respond to these therapeutic agents, providing alternative therapies for TP53-mutant patients. Importantly, the Millenium company has recently reached Phase III clinical trials with the AURKA inhibitor MLN8237 for the treatment of haematological and solid tumours. These kinases have roles in mitosis, a process deeply de-regulated in p53-mutant tumours [37]. In addition, both AURKA and PLK1 are directly regulated by p53 (Fig. S8A). We suggest that the efficacy of the inhibitors of these kinases in tumours overexpressing them probably depends on both the presence of p53 mutations and p53 pathway inhibition independent of TP53 mutation (as assessed by the 40-gene test). Thus, there are reports indicating that inhibitors to AURKA or to PLK display better efficacy with p53 mutation [38]–[40]. Using the Connectivity Map resource, we found that HDAC, mTOR, PIK3CA or topoisomerase II inhibitors might be beneficial for tumours with similar profile to our p53-deficient mouse carcinomas. Some of these compounds are being analyzed in clinical trials for cancer treatment, or already approved by the FDA (such as vorinostat for cutaneous T-cell lymphoma).

A number of genomic tests have been developed for human BC outcome based upon GE profiles, although only a small number have seen clinical implementation [41]. Since TP53 mutation is a predictor of poor prognosis, some of these BC tests based on GE are designed to predict TP53 mutational status [28], [31]. However, the 40-gene test discriminates poor outcome tumours with no TP53 mutation, demonstrating its greater sensitivity than mutational analysis in the detection of patients with low survival potential. This might be the consequence of other molecular alterations that produce p53 pathway inhibition being present, either in the upstream regulators or downstream effectors of p53.

The BC tumours produced in the MMTV-c-myc models show different degrees of similarity with the 682-gene signature, with about 50% of samples returning positive Pearson correlation values in the GSE15904 dataset (which contains 80 carcinomas). Although this model is poorly metastatic [42], [43], a signature of metastatic potential has been described in a subgroup of its tumour types [16]. Cooperation between p53 and c-myc may exist in p53^ΔEC^ and p53^ΔEC^;pRb^ΔEC^ skin carcinomas since they overexpress c-myc targets [12]. The close similarity of BC models owed to the expression of the SV40 T-antigen used in the C3(1)-Tag and WAP-TNP8 mice is due to the Large T-antigen deactivating p53 and pRb. These deactivations are also likely in human basal-like tumours since these are known to harbour p53 mutations [44], to have a high mitotic rate, and to show the greatest expression of proliferation genes, which are known E2F targets [45]. In addition, an interspecies comparison of mouse BC models and human BC samples has also shown strong similarities between SV40-derived models and human basal-like tumours at the genome-wide transcriptome level [46], confirming the present results. Finally, it has recently been described that a subgroup of carcinomas in the p53^fl/fl^;MMTV-cre transplant model, also with strongly 682-gene-like signatures, show marked enrichment in functional tumour-initiating cells in limiting dilution transplantation assays [47]. These findings further underscore the ESC characteristics of the p53-deficient skin carcinoma model.

The molecular and pathway changes that occur between primary carcinomas and metastases in the LAd Kras/Lkb1^L/L^ model have been associated with the enrichment of GE signatures associated with the ESC phenotype, and the activation of epithelial-mesenchymal-transition (EMT), focal adhesion and oncogenic signalling (EGFR or ERBB2) [11]. These associations agree with the present results: the p53^ΔEC^ and p53^ΔEC^;pRb^ΔEC^ skin models both show ESC signatures as well as the deregulation of EMT markers [12]. Whether the transition from primary to invasive tumours in the Kras/Lkb1^L/L^ model is facilitated by early mutations inhibiting the p53-dependent pathway or in the p53 alleles themselves remains to be determined. Carcinomas arising through Kras^G12D^ expression and homozygous p53 inhibition in the Kras^LA2/+^;Trp53^LSL/LSL^;Rosa26^CreERT2^ model showed a better correlation with 682-gene than with the Kras^G12D^ model (which has WT p53). This agrees with the reported high malignancy of double transgenic mouse tumours [48], [49].

The results of our retrospective validation of the BC 40-gene test in about 3000 patients from 12 different cohorts strongly suggests its clinical usefulness, although further validation involving prospective testing is required. Moreover, the validation showed this BC test to be independent of the microarray platform used in the datasets, as also seen for the LAd 36-gene predictor. However, for LAd, the number of GE-based datasets for outcome prediction testing was more limited. Nonetheless, LAd predictors are very necessary in early stage patients if the right form of clinical management is to be adopted. Here we show that the 36-gene genomic-clinical predictor to be of high sensitivity in terms of predicting good outcome in the patients in these validation cohorts, stratifying patients of all disease stages in terms of clinical outcome. Remarkably, this test also appeared to be of use with FFPE-samples/qRT-PCR, again providing good patient stratification. Further restrospective validation studies are necessary with larger numbers of patients.

In conclusion, the present results indicate that mouse skin carcinoma models with p53-deficiency show significant similarities to mouse BC and LAd models with functional inhibition of p53. These similarities can be exploited in the development of accurate predictors of human BC and LAd clinical outcome. Additional genomic testing to predict clinical behavior should be tried with other cancer types associated with p53-dependent malignancy. We already have preliminary data showing that predictors for prostate adenocarcinoma, multiple myeloma, and glioblastoma might be obtained.

## Materials and Methods

### Ethics Statement

The ethical committee of the Errikos Dunant Hospital in Athens (Greece) approved the research performed using FFPE blocks of carcinoma samples from lung cancer. Written informed consent has been obtained and the investigation has been conducted according to the principles expressed in the Declaration of Helsinki.

### Comparison of the 682-gene Signature in GEMMs of BC and LAd

GEMMs of BC and LAd for which genome-wide transcriptome analyses have been reported, and for which the raw data are publicly accessible, were used as models for comparison. To reduce artefactual similarities due to differences between microarray platforms, mouse datasets were selected from analyses performed using the same Affymetrix GeneChip with which the 682-gene signature was obtained (MOE 430 2.0), or other GeneChips that use the same probesets (MOE 430A, MOE 430A 2.0). A complete list of the GEMMs compared is described in Table S1 [10], [11], [16]–[23]. Raw data were downloaded from the GEO web site. Robust multichip average (RMA) [50], [51] was performed for each dataset in order to obtain log_2_-based signal intensity probeset values, from which z-scores (mean = 0, standard deviation = 1) were calculated. This standardization of the signal values allowed for direct comparison between different datasets in a single matrix. The T-values returned by Student’s t-test comparisons between normal skin and carcinoma samples in which the 682-gene signature was determined (GSE11990) were used to build a centroid template. The Pearson correlation coefficient (and the corresponding p-value) with respect to the centroid was calculated for each mouse sample. The mean correlation value was calculated for samples with similar genotypes and tissue types.

### Development of 40-gene and 36-gene Tests for Predicting the Clinical Outcome of Human Cancer

The genomic predictive tests were developed in a three-step procedure (Fig. S1). Step 1: Deregulated mouse p53-tumor genes (i.e., compared to normal skin tissue using our GSE11990 dataset) were selected to obtain the 682-gene signature as previously described [12]. Step 2: Cox regression analysis was performed for the human homologues of the mouse signature transcripts in a discovery human cancer dataset. Step 3: Patient p53 Risk Scores (p53RS) were calculated and receiver operating characteristic (ROC) curves analyses performed for selected gene groups based on Cox correlations with the clinical outcome.

Mapping from mouse to human was performed using the Ailun web tool (http://ailun.stanford.edu/) [52]. Affymetrix HG-U133A probesets of the human transcripts were used in subsequent similarity analyses. For BC, the Desmedt dataset was used as discovery dataset to obtain the predictor test (Table S2) [53]. Signal intensity values were obtained using RMA. Relative log expression (RLE) and normalised unscaled standard error (NUSE) plots allowed the identification of seven poor quality human cancer samples; these were discarded (final patient number = 191). Cox proportional hazard analysis was performed with the human homologues of the 682-signature, using censored distant metastasis (DM) at 5 years as the endpoint. A Cox hazard ratio of >0 is returned if the gene is overexpressed in prometastatic tissue, and below <0 if underexpressed. A Wald test [54], [55] was performed to check the null hypothesis of the coefficient being 0. Transcripts showing significant correlations with DM were assigned to probeset groups depending on their Wald values (S_i_): group i) S_i_ ≥3 or S_i_ ≤−3, group ii) S_i_ ≥2.5 or S_i_ ≤−2.5, or group iii) S_i_ ≥2 or S_i_ ≤−2. A p53RS formula was then developed (Fig. S2A) to quantify the metastatic potential of each tumour, based on the expression values and the Wald value calculated for each transcript probeset in the Cox analysis. A similar means of obtaining risk scores for each patient has been previously reported for predicting the outcome of human BC [56]. The prediction capabilities of the probeset groups were checked using ROC curves for the discovery dataset, by calculating the AUC values or the specificity at 100% sensitivity (Fig. S2B and C). The 40-probeset group (hereafter referred to as the 40-gene test) (Table S3) was selected since it showed the best AUC (0.77, p-val = 1×10^−7^) and specificity (40.1) values. Univariate Cox regression analysis showed the p53RS value, as a continuous variable, to be very significantly correlated with DM (p-val = 1.49×10^−7^). The patients were stratified into three risk groups by dividing the dataset into six p53RS percentiles (from lower to higher values), with the 1^st^ percentile for low risk, the 2^nd^ to 3^rd^ percentiles for intermediate risk, and the 4^th^ to 6^th^ percentiles for high risk. The corresponding p53RS threshold values defining each risk group were: low <−89.27; −89.27≤ intermediate <25.33; and high ≥25.33.

LAd samples from early stage patients (stages IA and IB) (n = 275) in the Shedden dataset [30] (Table S4) were used to develop the genomic predictor following the same three-step procedure described above (Fig. S1). Later disease stage samples were excluded from the discovery dataset since the accuracy of the genomic-based prediction dropped significantly when they were included (later stage patients have usually undergone aggressive treatments, which might affect the results). All discovery samples met the RLE and NUSE thresholds after RMA normalization. This dataset included 443 patients from four institutions. Overall survival (OS) at 3 years was used as the endpoint in Cox regression analysis of the human-mapped 682-gene transcripts. Probeset groups showing significant associations with OS were constructed based on Wald threshold similarities, as above. The same approach was then used to calculate the p53RS values for each patient (Fig. S2A). AUC and specificity values at 80% sensitivity (Fig. S2D and E) were obtained, providing an optimal 36-probeset group (hereafter referred to as the 36-gene test) (Table S5). The sensitivity selected was the highest possible in order to obtain a minimum of 40% specificity. A predictor based on clinical variables was established using data for all patients from two institutions represented in the Shedden dataset (HLM and MI) (n = 254). Cox analysis was then performed using age, gender and disease stage data since these variables are known to be correlated with clinical outcome [30]. Before calculation, gender and disease stage were coded using numerical values as follows: female = −1, male = +1; and stage IA = 1, IB = 2, and stage II or later = 3. Age was deemed to be a continuous variable. Z-scores were also calculated for each variable. After Cox analysis involving OS at 3 years, a clinical risk score (CRS) was calculated for each patient based on the Wald value for each clinical variable and the corresponding z-score value for each patient (Fig. S5A and Table S7). ROC analysis showed a significant association between CRS and OS (AUC = 0.66, p-val = 3×10^−4^). A combination of both the 36-gene genomic and clinical tests (36-gene genomic-clinical predictor) improved the overall accuracy within the combined dataset (n = 373) (AUC = 0.73, p-val = 9×10^−13^). This combination was established by adding the p53RS and the CRS values (Global Risk Score or GRS). Patient stratification was performed as for BC, by dividing the dataset into six percentiles of GRS (from lower to higher values): the 1^st^ percentile for low risk, the 2^nd^ and 3^rd^ percentiles for intermediate risk, and the 4^th^ to 6^th^ percentiles for high risk. The corresponding 36-gene genomic-clinical standardised threshold values defining each risk group were: low <−1.051; −1.051≤ intermediate <−0.098; and high ≥−0.098.

### Validation of the 40-gene Test as a Predictor of BC Clinical Outcome

The test datasets used to validate the predictive genomic test are described in Table S2 and elsewhere [28], [56]–[66]. They represent a total number of 2993 individual BC samples, 12 different datasets, 2 microarray platforms, and 4 different endpoints. p53RS was calculated independently in each dataset. For the Affymetrix datasets, signal intensity values were obtained using RMA, and the p53RS for each patient calculated using the 40 probesets. Risk groups were defined using the above-described thresholds. For the van de Vijver dataset (Agilent), gene annotations for the array used (GEO identifier GPL2567) were obtained using the Ailun web tool. Log_10_−based expression values were transformed to log_2_ values. Entrez IDs for the 40 probesets (32 total IDs) were extracted from the dataset, together with the log_2_ values for each sample. Thirty two Agilent probes were obtained corresponding to 27 unique Entrez genes. p53RS was obtained as follows: i) The mean Wald value of genes with more than one Affymetrix probeset was calculated, such that each gene was associated with a single final Wald value; ii) genes with more than one Agilent probe were independently multiplied by the Wald value of the corresponding gene. Absolute p53RS values were then calculated from the above 27 genes. To compare with Affymetrix values and divide the patients into intermediate, high and low risk groups, z-scores were calculated for the p53RS values for the patients in the van de Vijver dataset (Agilent) and the Desmedt discovery dataset (Affymetrix). The use of z-scores allows equivalence to be established between datasets with respect to the thresholds separating the risk groups.

### Validation of the 36-gene Genomic-clinical Test as a Predictor of LAd Clinical Outcome

The test datasets used to validate the predictive genomic-clinical test are described in Table S4. The LAd microarray datasets used included the remaining 67 samples from the Shedden dataset, 129 samples from the Nguyen dataset [67], and 117 samples from the Tomida dataset [68], representing a total number of 313 samples, and 2 different platforms (Affymetrix and Agilent). GRS calculation was performed independently for each dataset. For the Affymetrix datasets, signal intensities were obtained using RMA. For the Tomida dataset (Agilent), a normalised expression dataset was downloaded from the GEO website (GSE13213). The p53RS for each patient was calculated using the 36 probesets, and the CRS calculated using clinical variables. Risk groups were defined using the above-described thresholds for GRS. For the Tomida dataset, gene annotations for the array used (GEO identifier GPL6480) were obtained using the Ailun web tool. Entrez IDs for the 36 probesets (30 total IDs) were extracted from the dataset, together with the log_2_ values for each sample. Forty seven Agilent probes were obtained, corresponding to all 30 Entrez genes. The p53RS values were obtained as described for the 40-gene test. Since the GRS was calculated from the z-values for p53RS and CRS, no inter-platform transformation of absolute p53RS values was necessary. The same thresholds are therefore valid for both platforms.

### Validation of the 36-gene Genomic-clinical Test by qRT-PCR Using FFPE LAd Samples

Validation of the 36-gene genomic-clinical predictor was performed in FFPE-samples from 55 patients with LAd (Table S4). Briefly, the cancerous tissue on 4–5 slides (4 µm sections) per patient was dissected out. These were then deparaffinated using deparaffinisation solution (Qiagen). RNA extraction was performed using the miRNeasy FFPE Kit (Qiagen), which includes a DNase treatment step to avoid cellular DNA contamination. The RNA concentration of the FFPE samples was determined using a NanoDrop® ND-1000 UV-Vis Spectrophotometer (NanoDrop Technologies, Wilmington, DE, USA). cDNA synthesis was performed with oligonucleotide primers specific for 30 genes within the 36-gene signature, plus 6 housekeeping genes (ACTB, GAPDH, GUSB, RPLP0, TBP and TFRC), using the Omniscript RT Kit (Qiagen). qRT-PCR was performed with primers located upstream of the corresponding cDNA-synthesis primers, using the *Power* SYBR® Green PCR Master Mix (Applied Biosystems). The sequences of all primers used are detailed in Table S8.

qRT-PCR data normalisation was performed as previously described [69] with some modifications. Briefly, the mean of the housekeeping GE values was used as a reference, and a Ct value for the 36-gene test defined for each sample. To calculate the p53RS, the mean of the normalized GE values of the 30 non-housekeeping genes was determined, as illustrated in Figure S5B. The mean value for the housekeeping genes was very similar between samples. Six samples showing no housekeeping gene expression, or a low Ct value, were excluded from analysis. PCR-p53RS values were standardized and the z-values combined with those of the CRS as described above to obtain a GRS. The threshold PCR-p53RS values used to stratify the patients were the same as those used in the quantification of microarray-based GE. Due to the small number of samples (n = 55), the intermediate and high risk patients were combined to form a single group.

### Overlapping between the 682-gene Signature and the Transcriptome of Human Cancer Samples Sensitive to Targeting Therapies

Overexpressed and underexpressed mouse genes within the 682-gene signature in the p53-deficient carcinomas were mapped to human genes using the Ailun web tool and loaded into the Oncomine™/Compendia database [70]. The association of the mapped genes with the concepts of “Drug Sensitivity” or “Patient Treatment Response” was tested using Fisher’s exact test. Significance was set at Odds Ratio ≥2 and P≤0.0001. We selected for significant associations between the 682-gene signature and the transcriptomes of cancer samples sensitive to therapies targeting genes represented within the 40-gene signature. Alternatively, the overexpressed and underexpressed mouse genes within the 682-gene, mapped to human genes, were loaded into the Connectivity Map resource [34] (http://www.broadinstitute.org/cmap/). In brief, this resource consists of pattern-matching software that compares an input gene signature to a database of signatures from 164 small molecule bioactive compounds (dubbed perturbagens) (85 of which are classified as pharmaceutical drugs) and 4 cell lines: MCF7 (breast cancer), PC3 (prostate cancer), HL60 (leukemia), SKMEL5 (melanoma). A connectivity score from −1 to +1 is assigned based on the degree of similarity or dissimilarity between the two signatures. Thus, a drug with a low connectivity score has a gene signature very dissimilar to the query signature and might be hypothesized to inhibit a pathway in parallel with the transcription factor that generated the query signature. Results of permutation analyses were used, in which an estimate of the likelihood that the enrichment of a set of instances in the list of all instances in a given result would be observed by chance. This value is determined empirically by computing the enrichment of one hundred thousand sets of instances selected at random from the set of all instances in the result.

## Supporting Information

Figure S1 **Three-step procedure for obtaining the 40-gene and 36-gene predictors of clinical outcome.** **Step 1**: Signatures were obtained after expression profiling of mouse skin primary tumours and normal tissue. Mouse genes were mapped to human genes. **Step 2**: Cox regression analysis was performed to select tumour genes showing significant associations with survival in human BC and LAd discovery datasets. **Step 3**: Subgroups of probesets were independently tested as predictors using the p53 risk score (p53RS) formula and receiver operator curve (ROC) analysis within the corresponding discovery datasets (Fig. S2).
(TIF)

Figure S2 **Development of the breast and lung cancer outcome predictor tests.** **A**) p53 Risk Score (p53RS) formula. A risk score was calculated for each patient based on the Wald statistic and the log_2_ expression value for each probeset in the discovery dataset. Receiver operator curve (ROC) analysis was performed for selected gene groups of different size to calculate the prediction variables of i) area under the curve (AUC) for breast cancer (**B**), the AUC for lung adenocarcinoma (**D**), ii) and specificity at 100% sensitivity for breast cancer (**C**), or at 80% sensitivity for lung cancer **(E)**. See Materials and Methods for a detailed explanation.
(TIF)

Figure S3 **Kaplan-Meier curves for patients with BC and DMFS in eight datasets.** Patients were stratified as of low (green), intermediate (blue) or high (red) risk using the 40-gene test. Numbers at the left of each plot represent the number of patients within each risk group. p-val: significance of survival differences (log-rank test).
(TIF)

Figure S4 **Stratification and survival of patients with breast cancer using the 40-gene test and different endpoints.** **A**) Relapse-free survival in the pooled datasets (n = 978). **B**) Disease-free survival in the pooled datasets (n = 395). **C**) Overall survival in the pooled datasets (n = 781). Patients are stratified as being at low (green), intermediate (blue) and high (red) risk. p-val: significance of survival differences (log-rank test).
(TIF)

Figure S5 **A**) Formula for calculating human lung adenocarcinoma risk based on clinical variables. **B**) Formula for calculating human lung adenocarcinoma risk based on the 36-gene test (using qRT-PCR and FFPE-samples).
(TIF)

Figure S6 **Kaplan-Meier curves of OS for LAd patients in three datasets.** Patients were stratified as of low (green), intermediate (blue) or high (red) risk using the 36-gene genomic-clinical test. Survival plots are shown for patients of all stages (**A**, **B** and **C**) and early stage patients (**D** and **E**). The validation patients from the Shedden dataset do not include early stage patients. p-val: significance of survival differences (log-rank test).
(TIF)

Figure S7 **The expression of the genes represented by the 40-gene and 36-gene signatures is dependent on p53-expression; their expression is different in mouse models of mammary and lung carcinoma respectively.** The same is seen in human cancer breast and lung samples. A) Heatmap of the 40-gene signature transcripts from normal mammary gland (green) and breast carcinoma (red) from WAP-TNP8 transgenic mice. The middle samples, showing intermediate expression, include mammary glands from transgenic mice at 1, 2, 3, 4 and 5 months after the induction of the SV40-transgene (and subsequent p53 inhibition) (blue bars). Wald>0: genes overexpressed in high risk breast cancer patients. Wald<0: genes underexpressed in high risk breast cancer patients. B) Heatmap of the 36-gene signature transcripts from normal lung (green) and lung adenocarcinomas (red) from Kras^LA2/+^;Trp53^LSL/LSL^;Rosa26^CreERT2^ transgenic mice. The tamoxifen-induction p53 expression in adenocarcinomas is shown in the middle samples, which show intermediate expression. Wald>0: genes overexpressed in high risk lung adenocarcinoma patients. Wald<0: genes underexpressed in high risk lung adenocarcinoma patients.
(TIF)

Figure S8 **Inhibitory regulation by p53 (and/or pRb) of genes overexpressed in high risk tumours as determined by the 40-gene (A) and 36-gene (C) tests is known to occur, validating the essential role of the p53 pathway in repressing these genes.** Genes within the 40-gene (B) and 36-gene (D) signatures activate cell proliferation, and inhibit cell differentiation and apoptosis. Genes in red are overexpressed in high risk tumours; genes in blue are underexpressed in high risk tumours. Red lines: direct inhibition between gene products or cellular processes. Green lines: direct activation between gene products or cellular processes. Dashed lines: regulation not demonstrated to be direct. Numbers close to coloured lines: number of PubMed publications citing interactions between gene products (panels A and B) or biological processes (panels C and D). Analysis performed using Pathway Studio® software from Ariadne Genomics.
(TIF)

Figure S9 **p53 mutation biomarkers within the 40-gene signature as alternative targets for cancer treatment.** **A**) Response to the AURKA inhibitor MLN8237 was tested in human cancer xenografts. Comparison of the transcriptomes of responder and non-responder samples with the 682-gene signature. Significant transcript overlapping was observed for overexpressed genes in both the mouse signature and the responders. The number of responder/non-responder human tumours is shown, as is the number of common genes, and the level of significance for overlapping (p-val) using Fisher’s exact test. **B**) Sensitivity to inhibitors of AURKB and PLK1 was tested in a collection of human cancer cell lines. Comparison of the transcriptomes of the sensitive and resistant lines with the 682-gene signature. Significant transcript overlapping was observed for overexpressed genes in both the mouse signature and the sensitive cells. For each inhibitor, the number of sensitive/resistant cell lines is shown, as is the number of common genes, and the level of significance for overlapping (p-val).
(TIF)

Figure S10 **Survival curves for BC patients as predicted by the 40-gene test and the p53 mutation genomic test.** **A**) Comparison of patient stratification by the 40-gene and p53 mutation status predictor tests, performed with the Miller BC dataset. Survival curves for the same patients produced by both stratification methods are shown. **B**) Combination of the 40-gene test and p53 mutation status predictor test in the Miller BC dataset. Patients are grouped in p53-WT-pred low, intermediate and high risk groups, and p53-MUT-pred low, intermediate and high risk groups. p-val: significance of survival differences (log-rank test).
(TIF)

Table S1 **Mouse mammary and lung adenocarcinoma models used to validate the 682-gene signature.**
(XLS)

Table S2 **Gene expression datasets for human breast cancer with clinical information.**
(XLS)

Table S3 **Probesets corresponding to the 40-gene test, and associated Cox analysis.**
(XLS)

Table S4 **Gene expression datasets for human lung adenocarcinoma with clinical information.**
(XLS)

Table S5 **Probesets corresponding to the 36-gene test, and associated Cox analysis.**
(XLS)

Table S6 **Multivariate Cox regression analysis for the 40-gene test and p53 mutation status test.**
(XLS)

Table S7 **Clinical variables in the lung adenocarcinoma clinical test, and associated Cox regression analysis.**
(XLS)

Table S8 **Primer sequences used for qRT-PCR validation of the 36-gene test using FFPE-samples from lung adenocarcinoma patients.**
(XLS)

Table S9 **Molecular annotations of the clinical breast cancer specimens with respect to ER, PR and HER2 status.**
(XLS)

Table S10 **Molecular annotations of the clinical lung adenocarcinoma specimens with respect to EGFR and KRAS mutational status.**
(XLS)
