# Supplementary figures and images for: Mouse p53-Deficient Cancer Models as Platforms for Obtaining Genomic Predictors of Human Cancer Clinical Outcomes
Source: PLoS One. 2012 Aug 7;7(8):e42494. doi: 10.1371/journal.pone.0042494 (PMC3413665; doi:10.1371/journal.pone.0042494)

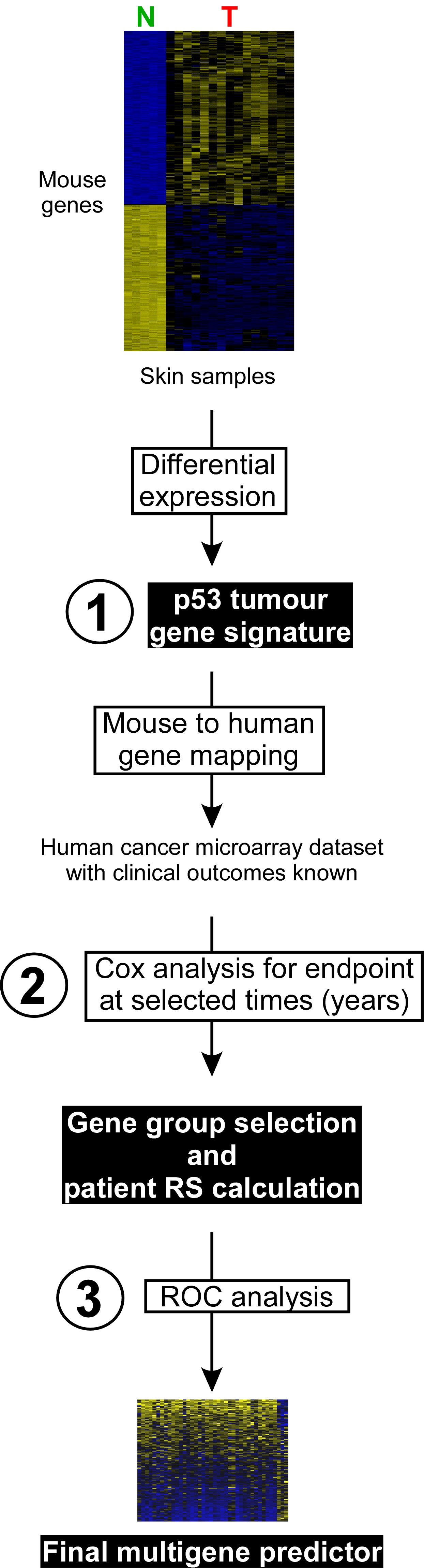

Supplement: Figure S1 — Three-step procedure for obtaining the 40-gene and 36-gene predictors of clinical outcome. Step 1: Signatures were obtained after expression profiling of mouse skin primary tumours and normal tissue. Mouse genes were mapped to human genes. Step 2: Cox regression analysis was performed to select tumour genes showing significant associations with survival in human BC and LAd discovery datasets. Step 3: Subgroups of probesets were independently tested as predictors using the p53 risk score (p53RS) formula and receiver operator curve (ROC) analysis within the corresponding discovery datasets (Fig. S2). (TIF) [file pone.0042494.s001.tif]

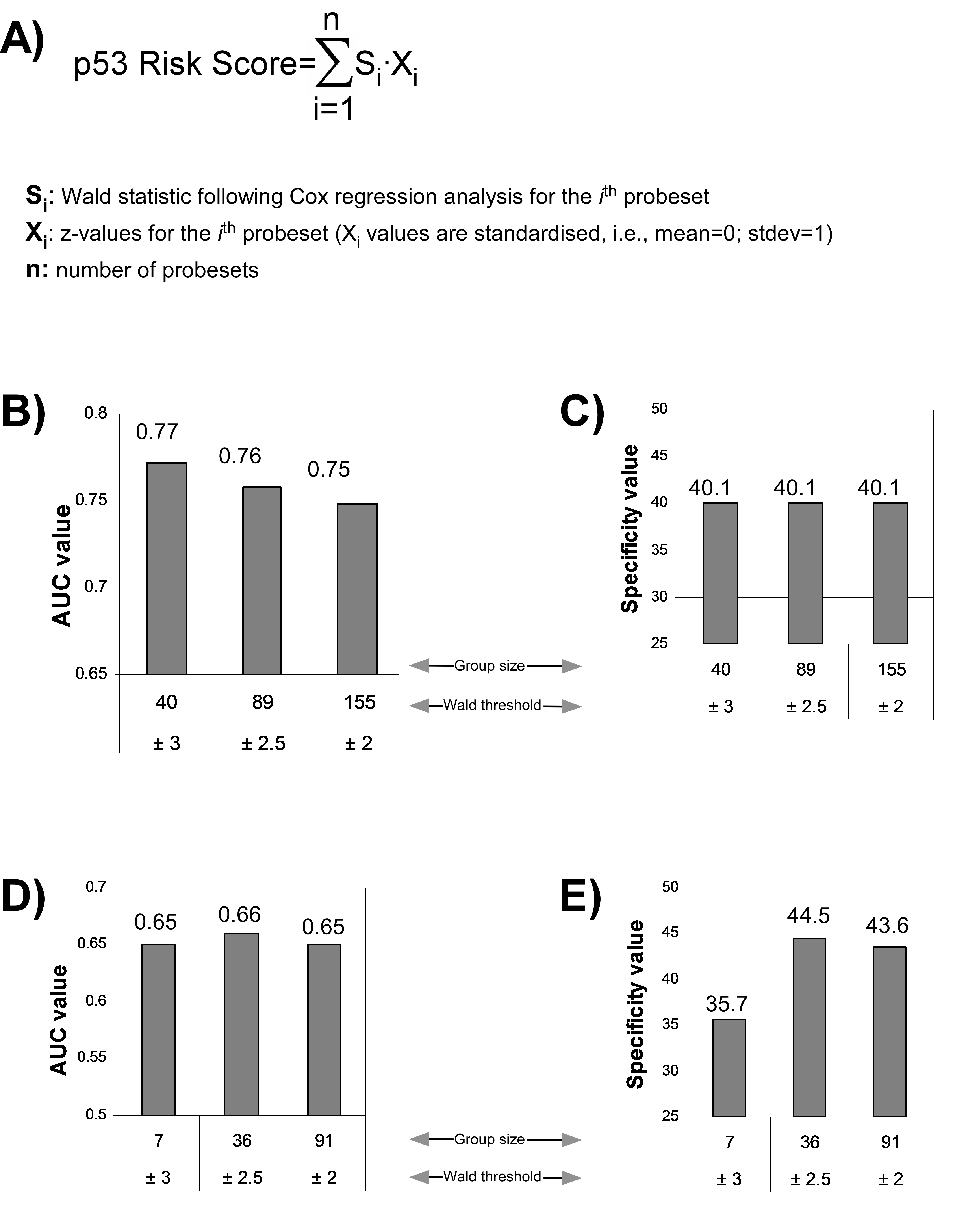

Supplement: Figure S2 — Development of the breast and lung cancer outcome predictor tests. A) p53 Risk Score (p53RS) formula. A risk score was calculated for each patient based on the Wald statistic and the log2 expression value for each probeset in the discovery dataset. Receiver operator curve (ROC) analysis was performed for selected gene groups of different size to calculate the prediction variables of i) area under the curve (AUC) for breast cancer (B), the AUC for lung adenocarcinoma (D), ii) and specificity at 100% sensitivity for breast cancer (C), or at 80% sensitivity for lung cancer (E). See Materials and Methods for a detailed explanation. (TIF) [file pone.0042494.s002.tif]

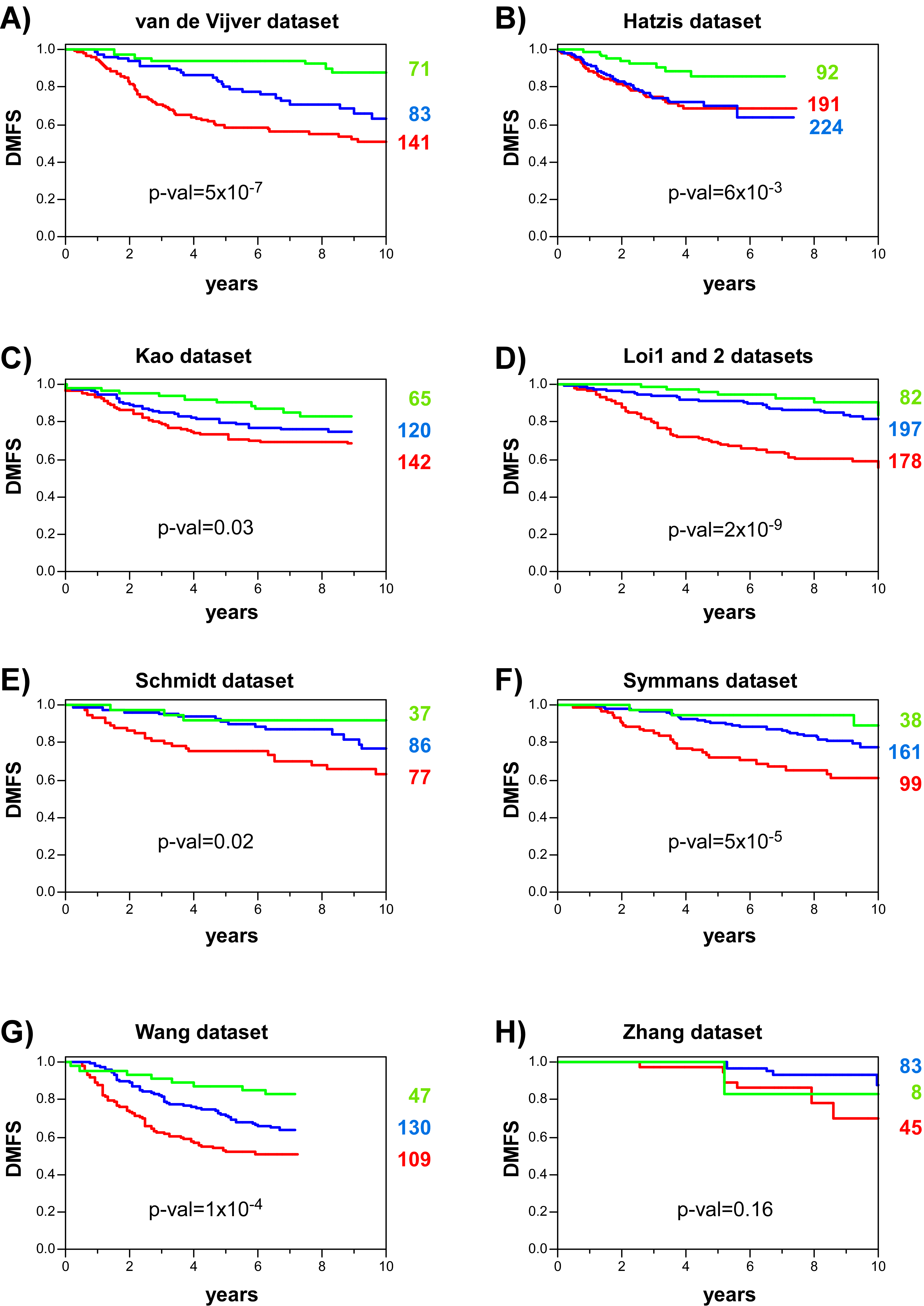

Supplement: Figure S3 — Kaplan-Meier curves for patients with BC and DMFS in eight datasets. Patients were stratified as of low (green), intermediate (blue) or high (red) risk using the 40-gene test. Numbers at the left of each plot represent the number of patients within each risk group. p-val: significance of survival differences (log-rank test). (TIF) [file pone.0042494.s003.tif]

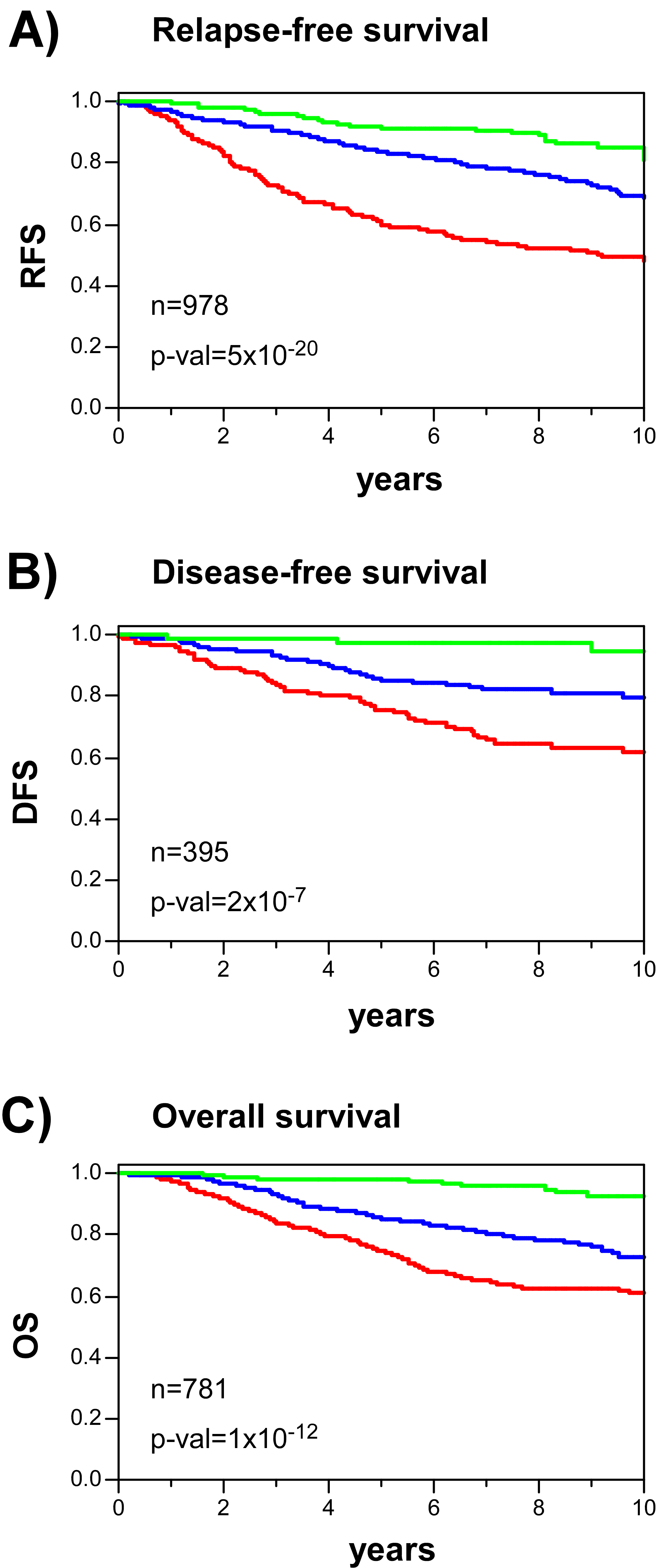

Supplement: Figure S4 — Stratification and survival of patients with breast cancer using the 40-gene test and different endpoints. A) Relapse-free survival in the pooled datasets (n = 978). B) Disease-free survival in the pooled datasets (n = 395). C) Overall survival in the pooled datasets (n = 781). Patients are stratified as being at low (green), intermediate (blue) and high (red) risk. p-val: significance of survival differences (log-rank test). (TIF) [file pone.0042494.s004.tif]

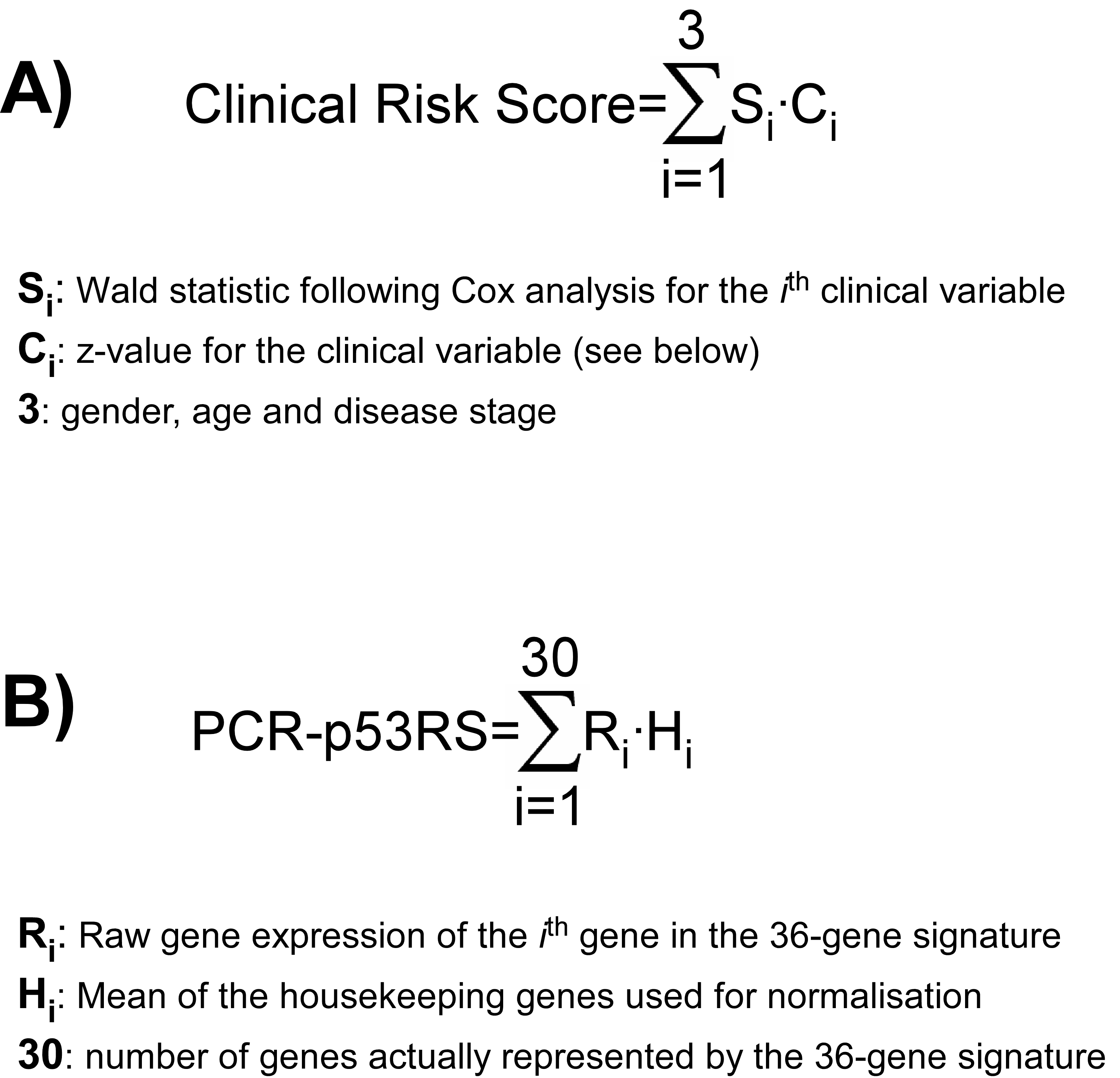

Supplement: Figure S5 — A) Formula for calculating human lung adenocarcinoma risk based on clinical variables. B) Formula for calculating human lung adenocarcinoma risk based on the 36-gene test (using qRT-PCR and FFPE-samples). (TIF) [file pone.0042494.s005.tif]

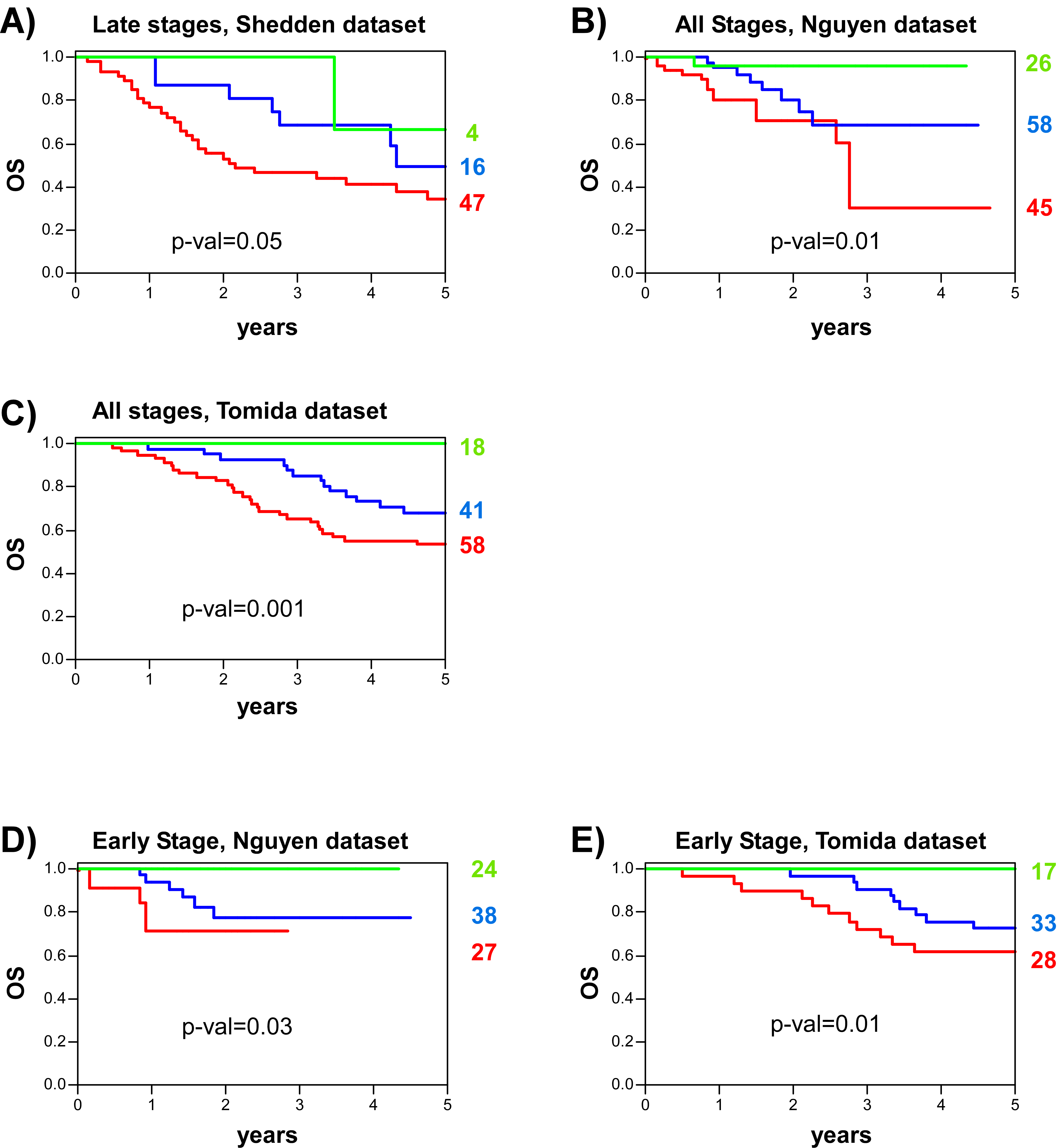

Supplement: Figure S6 — Kaplan-Meier curves of OS for LAd patients in three datasets. Patients were stratified as of low (green), intermediate (blue) or high (red) risk using the 36-gene genomic-clinical test. Survival plots are shown for patients of all stages (A, B and C) and early stage patients (D and E). The validation patients from the Shedden dataset do not include early stage patients. p-val: significance of survival differences (log-rank test). (TIF) [file pone.0042494.s006.tif]

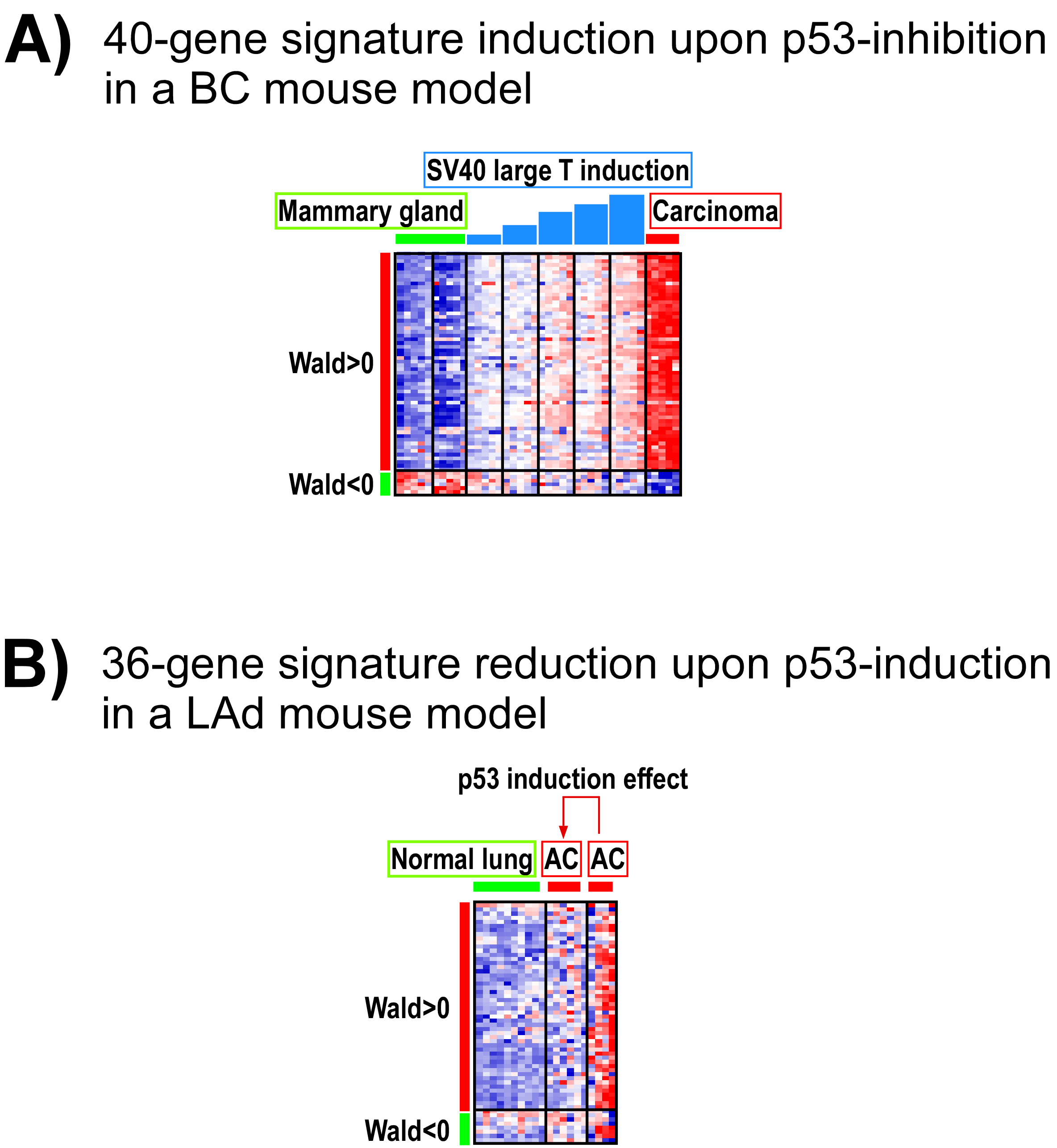

Supplement: Figure S7 — The expression of the genes represented by the 40-gene and 36-gene signatures is dependent on p53-expression; their expression is different in mouse models of mammary and lung carcinoma respectively. The same is seen in human cancer breast and lung samples. A) Heatmap of the 40-gene signature transcripts from normal mammary gland (green) and breast carcinoma (red) from WAP-TNP8 transgenic mice. The middle samples, showing intermediate expression, include mammary glands from transgenic mice at 1, 2, 3, 4 and 5 months after the induction of the SV40-transgene (and subsequent p53 inhibition) (blue bars). Wald>0: genes overexpressed in high risk breast cancer patients. Wald<0: genes underexpressed in high risk breast cancer patients. B) Heatmap of the 36-gene signature transcripts from normal lung (green) and lung adenocarcinomas (red) from KrasLA2/+;Trp53LSL/LSL;Rosa26CreERT2 transgenic mice. The tamoxifen-induction p53 expression in adenocarcinomas is shown in the middle samples, which show intermediate expression. Wald>0: genes overexpressed in high risk lung adenocarcinoma patients. Wald<0: genes underexpressed in high risk lung adenocarcinoma patients. (TIF) [file pone.0042494.s007.tif]

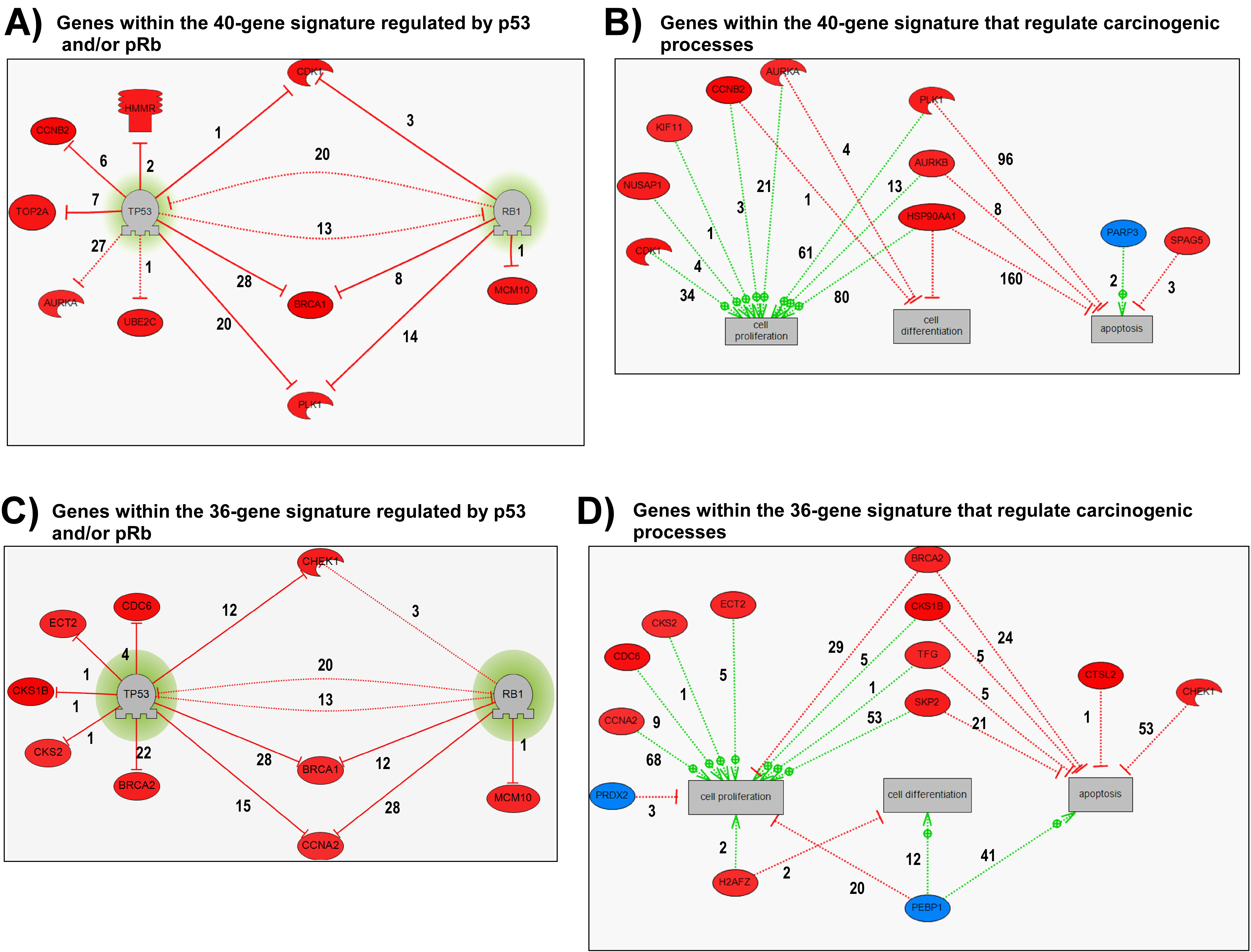

Supplement: Figure S8 — Inhibitory regulation by p53 (and/or pRb) of genes overexpressed in high risk tumours as determined by the 40-gene (A) and 36-gene (C) tests is known to occur, validating the essential role of the p53 pathway in repressing these genes. Genes within the 40-gene (B) and 36-gene (D) signatures activate cell proliferation, and inhibit cell differentiation and apoptosis. Genes in red are overexpressed in high risk tumours; genes in blue are underexpressed in high risk tumours. Red lines: direct inhibition between gene products or cellular processes. Green lines: direct activation between gene products or cellular processes. Dashed lines: regulation not demonstrated to be direct. Numbers close to coloured lines: number of PubMed publications citing interactions between gene products (panels A and B) or biological processes (panels C and D). Analysis performed using Pathway Studio® software from Ariadne Genomics. (TIF) [file pone.0042494.s008.tif]

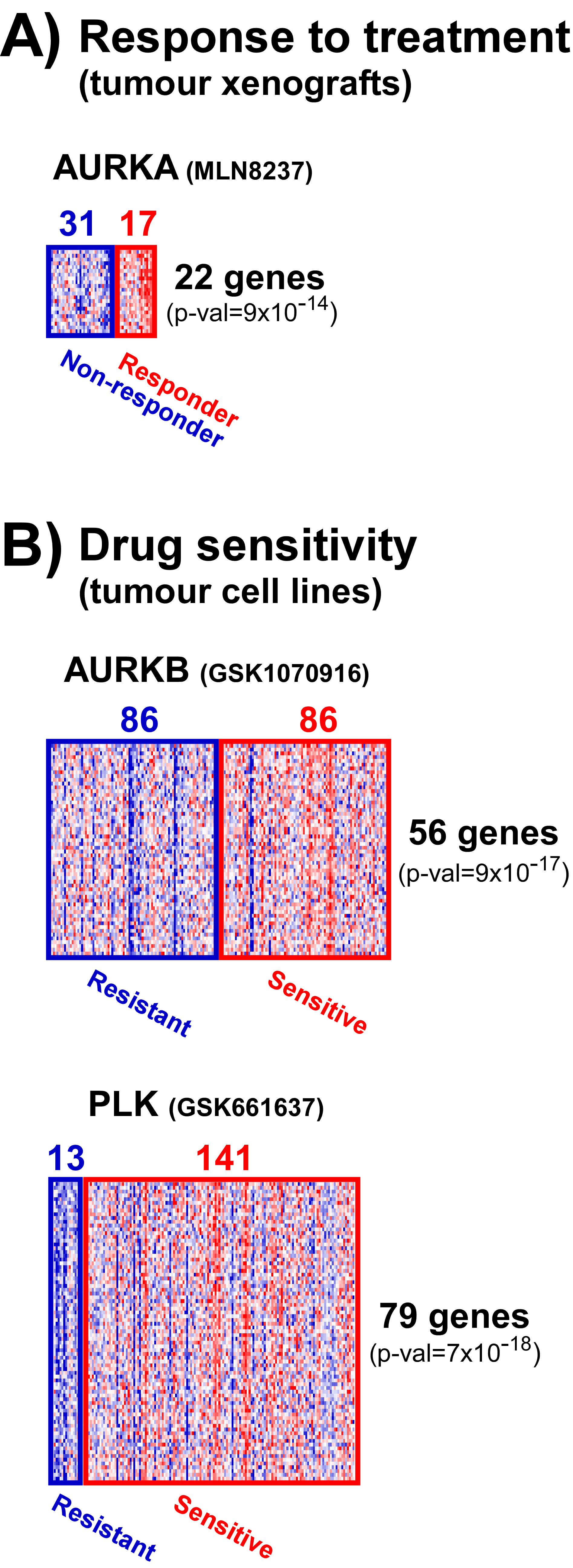

Supplement: Figure S9 — p53 mutation biomarkers within the 40-gene signature as alternative targets for cancer treatment. A) Response to the AURKA inhibitor MLN8237 was tested in human cancer xenografts. Comparison of the transcriptomes of responder and non-responder samples with the 682-gene signature. Significant transcript overlapping was observed for overexpressed genes in both the mouse signature and the responders. The number of responder/non-responder human tumours is shown, as is the number of common genes, and the level of significance for overlapping (p-val) using Fisher’s exact test. B) Sensitivity to inhibitors of AURKB and PLK1 was tested in a collection of human cancer cell lines. Comparison of the transcriptomes of the sensitive and resistant lines with the 682-gene signature. Significant transcript overlapping was observed for overexpressed genes in both the mouse signature and the sensitive cells. For each inhibitor, the number of sensitive/resistant cell lines is shown, as is the number of common genes, and the level of significance for overlapping (p-val). (TIF) [file pone.0042494.s009.tif]

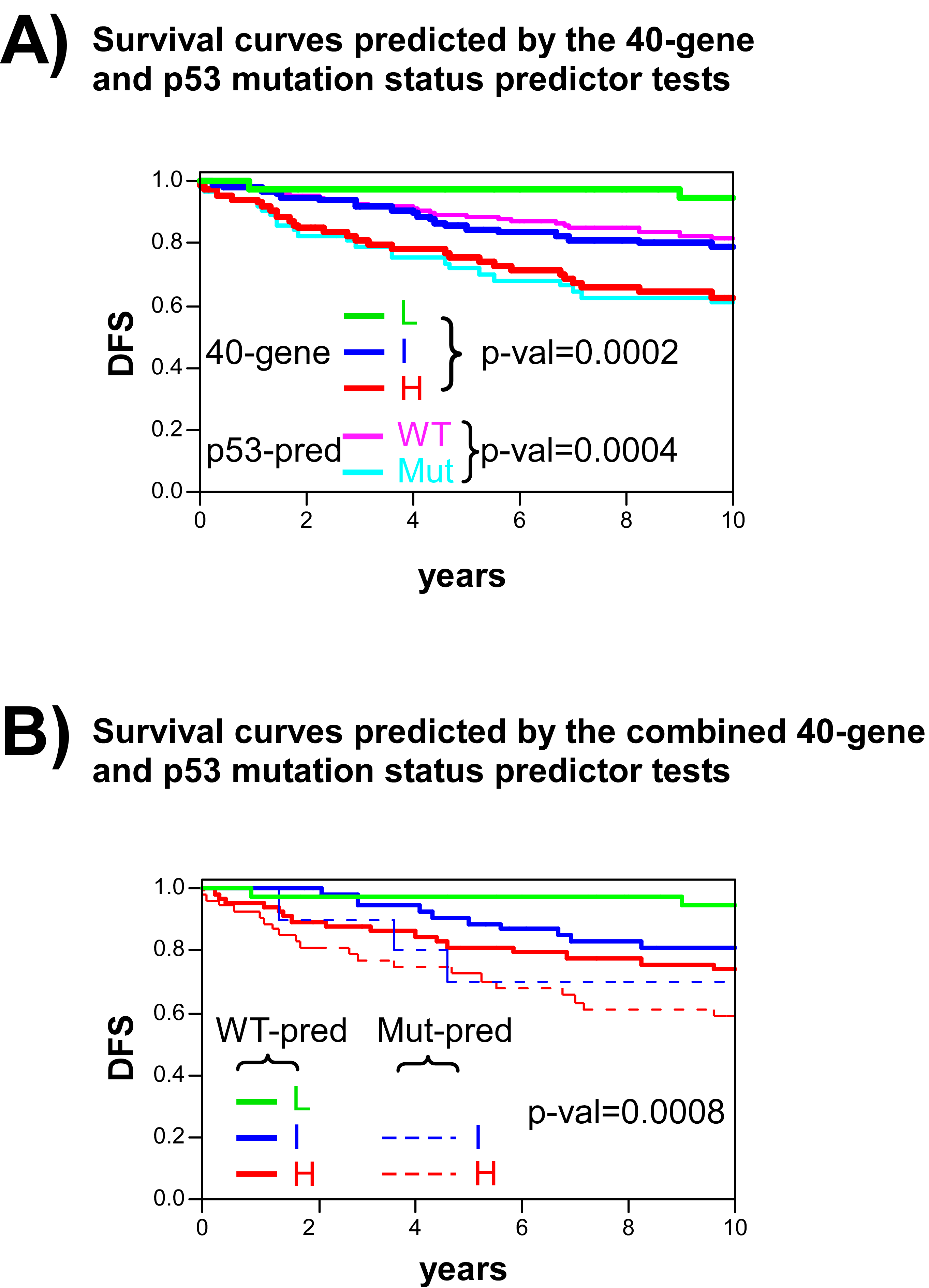

Supplement: Figure S10 — Survival curves for BC patients as predicted by the 40-gene test and the p53 mutation genomic test. A) Comparison of patient stratification by the 40-gene and p53 mutation status predictor tests, performed with the Miller BC dataset. Survival curves for the same patients produced by both stratification methods are shown. B) Combination of the 40-gene test and p53 mutation status predictor test in the Miller BC dataset. Patients are grouped in p53-WT-pred low, intermediate and high risk groups, and p53-MUT-pred low, intermediate and high risk groups. p-val: significance of survival differences (log-rank test). (TIF) [file pone.0042494.s010.tif]
